# Convergent olfactory trace amine-associated receptors detect biogenic polyamines with distinct motifs *via* a conserved binding site

**DOI:** 10.1016/j.jbc.2021.101268

**Published:** 2021-09-30

**Authors:** Liang Jia, Shengju Li, Wenxuan Dai, Lingna Guo, Zhengrong Xu, Anne M. Scott, Zhe Zhang, Jianfeng Ren, Qinghua Zhang, Thomas S. Dexheimer, Yu-Wen Chung-Davidson, Richard R. Neubig, Qian Li, Weiming Li

**Affiliations:** 1 Center for Brain Science, Shanghai Children’s Medical Center, Shanghai Jiao Tong University School of Medicine, Shanghai, China; 2 Department of Anatomy and Physiology, Shanghai Jiao Tong University School of Medicine, Shanghai, China; 3 Department of Fisheries and Wildlife, Michigan State University, East Lansing, Michigan, USA; 4 College of Fisheries and Life Sciences, Shanghai Ocean University, Shanghai, China; 5 Department of Otolaryngology Head and Neck Surgery, Jiangsu Provincial Key Medical Discipline (Laboratory), Affiliated Drum Tower Hospital of Nanjing University Medical School, Nanjing, China; 6 National Center for Advancing Translational Sciences, National Institutes of Health, Rockville, Maryland, USA; 7 Department of Pharmacology and Toxicology, Michigan State University, East Lansing, Michigan, USA; 8 Shanghai Research Center for Brain Science and Brain-Inspired Intelligence, Shanghai, China

**Keywords:** olfaction, biogenic amine, polyamine, trace amine-associated receptor (TAAR), G-protein coupled receptor (GPCR), homology modeling, docking, structure–function, site-directed mutagenesis, ligand-recognition site, CAD, cadaverine, DMEM, Dulbecco’s modified eagle medium, EC_50_, half maximal effective concentration, E_max_, maximal efficacy, G_αolf_, olfactory G protein, GPCR, G-protein-coupled receptor, mRTPs, mouse receptor transporting proteins, mTAAR9, mouse trace amine-associated receptor 9, PUT, putrescine, SPD, spermidine, SPM, spermine, sTAAR365, sea lamprey trace amine-associated receptor 365, TAAR, trace amine-associated receptor, TEA, triethylamine, TR-FRET assay, time-resolved fluorescence energy transfer assay

## Abstract

Biogenic amines activate G-protein-coupled receptors (GPCRs) in the central nervous system in vertebrate animals. Several biogenic amines, when excreted, stimulate trace amine-associated receptors (TAARs), a group of GPCRs in the main olfactory epithelium, and elicit innate behaviors. How TAARs recognize amines with varying numbers of amino groups is largely unknown. We reasoned that a comparison between lamprey and mammalian olfactory TAARs, which are thought to have evolved independently but show convergent responses to polyamines, may reveal structural determinants of amine recognition. Here, we demonstrate that sea lamprey TAAR365 (sTAAR365) responds strongly to biogenic polyamines cadaverine, putrescine, and spermine, and shares a similar response profile as a mammalian TAAR (mTAAR9). Docking and site-directed mutagenesis analyses show that both sTAAR365 and mTAAR9 recognize the two amino groups of cadaverine with the conserved Asp^3.32^ and Tyr^6.51^ residues. sTAAR365, which has remarkable sensitivity for cadaverine (EC_50_ = 4 nM), uses an extra residue, Thr^7.42^, to stabilize ligand binding. These cadaverine recognition sites also interact with amines with four and three amino groups (spermine and spermidine, respectively). Glu^7.36^ of sTAAR365 cooperates with Asp^3.32^ and Thr^7.42^ to recognize spermine, whereas mTAAR9 recognizes spermidine through an additional aromatic residue, Tyr^7.43^. These results suggest a conserved mechanism whereby independently evolved TAAR receptors recognize amines with two, three, or four amino groups using the same recognition sites, at which sTAAR365 and mTAAR9 evolved distinct motifs. These motifs interact directly with the amino groups of the polyamines, a class of potent and ecologically important odorants, mediating olfactory signaling.

Biogenic amines are a group of signaling molecules that activate G-protein-coupled receptors (GPCRs) and regulate a wide variety of neurophysiologic and behavioral functions. Recognition of amine neurotransmitters, which are often monoamines that activate the aminergic family of GPCRs in vertebrate central nervous systems, has been examined extensively (1, 2, 3). Besides, some excreted biogenic amines function as odorants and are detected by another family of GPCRs, the olfactory trace amine-associated receptors (TAARs) (4, 5, 6, 7, 8, 9, 10). These molecules are categorized based on the number of amino groups as either monoamine (one amino group, such as tyramine, tryptamine, phenylethylamine, and triethylamine) or polyamine (two or more amino groups, such as putrescine, cadaverine, spermidine, and spermine). To date, the structural basis of a TAAR receptor recognizes amines with one or two amino groups having been examined (11, 12, 13). However, how TAAR receptors recognize polyamines with three or four amino groups has not been determined. Thus, exploring the mechanism whereby TAARs respond to polyamines with two, three, or four amino groups will complete the story on how biogenic amines with one through four amino groups are recognized by GPCRs.

Odorous polyamines are found in natural excretions (urine, feces, and semen), decomposed tissues, and food sources, and can elicit significant physiological changes and behavioral responses in various species examined (4, 5, 6, 7, 8, 9, 10, 11, 12, 13, 14, 15). Cadaverine and putrescine, the foul-smelling diamines produced by microbial metabolism of putrefied animal tissue, repel zebrafish by activating an olfactory TAAR receptor (zTAAR13c) (9). Similarly, cadaverine activates an olfactory TAAR receptor in mouse (mTAAR9) and elicits either neutral or aversive behavioral responses, depending on the particular behavioral paradigm (16, 17). In contrast, putrescine is attractive to mice, although the cognate receptor or receptors have not been identified (16). Also, both cadaverine and putrescine can elicit feeding behaviors in rat and goldfish (18, 19). In addition, spermine, an abundant polyamine in the semen of male sea lamprey, acts as a male sex pheromone that specifically attracts ovulated females (5). A sea lamprey TAAR receptor, sTAAR348, is proposed to play a key role in mediating the pheromone function of spermine (5). Likewise, spermine and spermidine (a biosynthetic precursor of spermine) activate mTAAR9 and elicit neutral or attractive behavioral preferences, respectively (16). However, the mechanisms for TAARs in recognizing polyamines have not been fully determined. We argue that vertebrate TAARs have retained a conserved mechanism for polyamine recognition, even though the behavioral responses to the polyamines are species-specific and context-dependent.

Olfactory TAAR gene families are present in all vertebrate species (20, 21). Phylogenetic analysis revealed that TAARs of sea lamprey (a jawless vertebrate) cluster into an independent clade that is distantly related to the TAAR clade of jawed animals (20, 21, 22). Given that sea lamprey and mouse TAARs both detect the same group of polyamines, this provides an excellent opportunity to study the functional convergence of two independently evolved TAAR subfamilies. Many olfactory TAARs retain amine recognition motifs that are conserved in classical aminergic receptors, including an aspartate residue in transmembrane helix III (Asp^3.32^; Ballesteros–Weinstein indexing) and a tryptophan residue in transmembrane helix VII (Trp^7.40^) (22, 23). Molecular docking and mutagenesis studies of mammalian TAAR1, mTAAR7e, and mTAAR7f demonstrate that the negatively charged residue Asp^3.32^ is critical for amine recognition and forms a salt bridge with the ligand amino group. Other highly variable residues in the transmembrane domains contribute to the selectivity for ligands and serve as scaffolds that stabilize ligand binding (12). By contrast, a large number of teleost-TAARs lack Asp^3.32^, and instead, use Asp^5.42^ to form a salt bridge with an amino group of the biogenic amine. Several TAARs such as zTAAR13c, contain both Asp^3.32^ and Asp^5.42^ and recognize dicationic molecules including cadaverine and putrescine (13). Notably, almost all sea lamprey and mouse TAARs have only a single negatively charged residue, Asp^3.32^ or Glu^3.32^ in transmembrane helix III that could theoretically recognize only one amino group of the polyamines. The structural basis for these TAARs to stabilize their interaction with the other amino groups in polyamines remains elusive. It is likely that vertebrate olfactory TAARs feature a salt bridge that engages a ligand amino group and have other scaffolds that contribute to the specificity of polyamine recognition. However, the structures of these predicted scaffolds and their function in recognizing amines with two or more amino groups have not been elucidated.

We hypothesized that vertebrate TAARs rely on residues that form a cation–pi interaction or a hydrogen bond with the amino groups in addition to the salt bridge formed by Asp^3.32^ to recognize polyamines. In this study, we identified a sea lamprey olfactory TAAR receptor (sTAAR365) that shows a strikingly similar response profile to cadaverine, putrescine, and spermine as does mTAAR9. Through a systematic comparison of these two distant receptors with convergent functions, we show that sTAAR365 and mTAAR9 both possess conserved Asp^3.32^ and Tyr^6.51^ residues that interact with the two amino groups in cadaverine. In addition, sTAAR365 uses an extra Thr^7.42^ that stabilizes the recognition of cadaverine, serving as part of the amine-binding motif. In sTAAR365, this motif uses an additional negatively charged residue Glu^7.36^ that cooperates with Asp^3.32^ and Thr^7.42^ to recognize the tetraamine spermine. Likewise, mTAAR9 recognizes the triamine spermidine through an aromatic residue, Tyr^7.43^. Thus, sTAAR365 and mTAAR9 recognize these polyamines through a novel motif located in the transmembrane α-helices VI and VII. Taken together, our results propose a mechanism that sTAAR365 and mTAAR9 converged on their polyamine recognition through distinct motifs in a conserved binding site.

## Results

### Mammalian TAAR9 orthologs recognize biogenic polyamines

**Figure 1:**
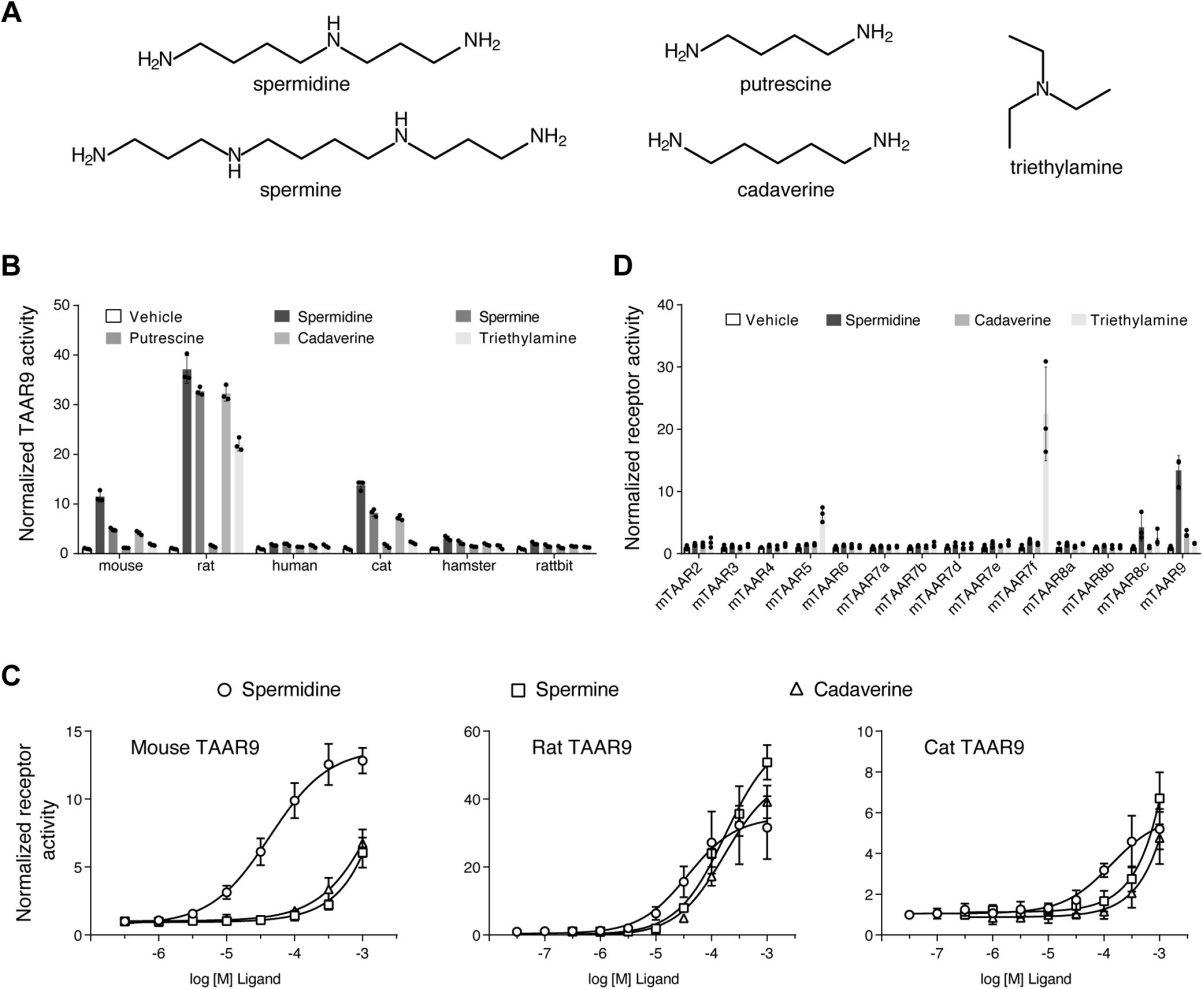
**Mammalian TAAR9s are broadly tuned to respond to various volatile amines.** Mammalian TAAR9 plasmids were transfected into Hana3A cells along with a cAMP-dependent luciferase reporter plasmid (CRE-Luc). Transfected cells were incubated with tested ligands, and luciferase activity was quantified with a fluorescent substrate as a reporter for G_olf_-mediated TAAR activation. Luciferase activity was indicated by the luminescence value and normalized as a fold-increase over the response to the vehicle stimuli (mean ± S.D., n = 3). *A*, chemical structures of the tested ligands spermidine, spermine, putrescine, cadaverine, and triethylamine. *B*, Hana3A cells expressing mouse, rat, human, cat, hamster, and rabbit TAAR9 were incubated with either vehicle or tested ligands (1 mM) and assayed for reporter activity. *C*, concentration-dependent luciferase activity in Hana3A cells expressing mouse, rat, and cat TAAR9 receptors stimulated with cadaverine, spermidine, and spermine. *D*, Hana3A cells expressing 14 mouse olfactory TAARs were incubated with vehicle, spermidine, cadaverine, or triethylamine (500 μM) and assayed for reporter activity.

We first asked whether mammalian TAAR9 orthologs are broadly tuned to triethylamine, cadaverine, spermidine, and spermine, as has been shown in mTAAR9 (16). We examined the response of TAAR9s from rat, human, hamster, and rabbit to the stimulation of 1 mM amines using a well-established cAMP response element (CRE)-driven luciferase reporter assay based on G_olf_-mediated cAMP signal transduction (5, 24). In addition to the four mTAAR9 ligands, we included putrescine, which is also a polyamine and precursor of spermine/spermidine biosynthesis. The tested TAAR9s were not activated by putrescine but exhibited varying degrees of activation to the other four amines (Fig. 1*B*). Rat TAAR9 showed the maximum responses to triethylamine, cadaverine, spermidine, and spermine compared with the other mammalian species. Cat TAAR9 displayed similar activation properties (potency and efficacy) to those of mTAAR9. These mTAAR9 ligands induced concentration-dependent activities in cells expressing mouse, rat, and cat TAAR9s (Fig. 1*C*). In contrast, human, hamster, and rabbit TAAR9s showed minimal activities for this set of ligands.

We then sought to determine if other mouse olfactory TAARs are activated by the mTAAR9 ligands. Only mTAAR8c showed modest activity to 500 μM spermidine (Fig. 1*D*). Triethylamine induced robust responses by mTAAR7f and moderate activities for mTAAR5 and mTAAR8c (Fig. 1*D*). Other TAARs were not activated by cadaverine or spermidine. Based on these findings, we concluded that several mammalian TAAR9 orthologs detect polyamines. As the mouse is a model animal for olfactory studies, we focused the remainder of our studies on mouse TAAR9 to further characterize TAAR interactions with polyamines.

### Sea lamprey olfactory sTAAR365 exhibits a similar polyamine response profile to mTAAR9

**Figure 2:**
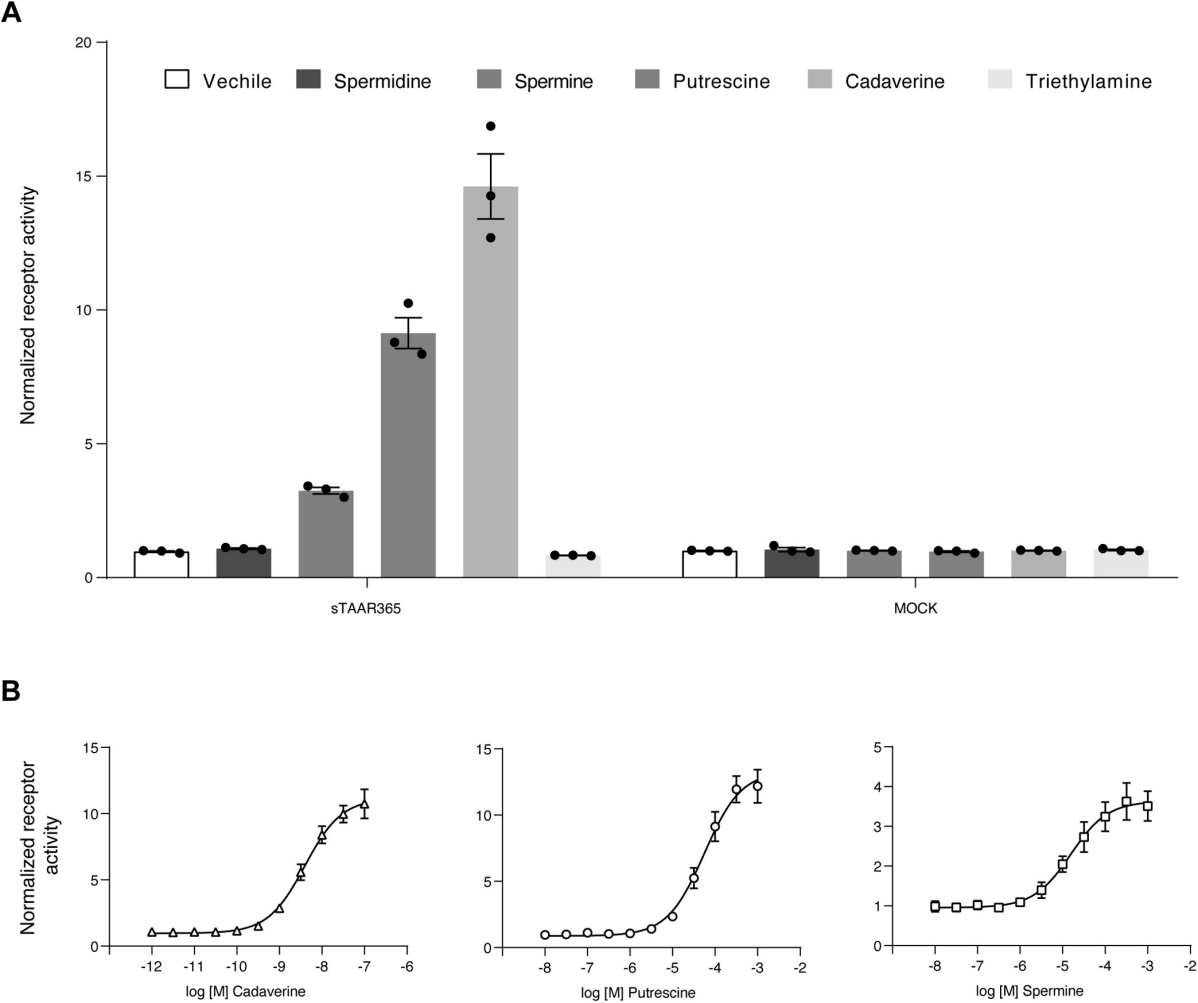
**Sea lamprey sTAAR365 exhibits similar response profiles as mammalian TAAR9.** HEK293T cells were transfected with sea lamprey sTAAR365 for 24 h and assayed for G_olf_-mediated cAMP production with a TR-FRET assay at 30 min after ligand addition. Receptor activity was normalized to the cAMP level in buffer-treated cells (mean ± S.D., n = 3). *A*, HEK293T cells expressing sTAAR365 or mock plasmid were incubated with either vehicle or tested ligands (1 mM). Spermine, putrescine, and cadaverine induced cAMP accumulation in HEK293T cells expressing sTAAR365. *B*, concentration-dependent cAMP production of HEK293T cells expressing sTAAR365 stimulated with cadaverine, putrescine, and spermine.

Next, we questioned whether the sea lamprey TAAR repertoire contains members that are broadly tuned to biogenic amines and that share similar response profiles with mTAAR9. In a previous study, we reported that sTAAR348 responds to spermine when expressed in HEK293T cells but not to other structurally related biogenic amines (5). Sequence alignment analyses indicated that sTAAR365 shares 74% sequence identity with sTAAR348 (Fig. S1). sTAAR348 and sTAAR365 showed 34.0% and 34.5% sequence identity, respectively, with mTAAR9 (Fig. S1). We reasoned that sTAAR365 could be a candidate as a polyamine receptor. To test this hypothesis, we used an established cAMP assay to examine the amine response properties of sTAAR365 (5). sTAAR365 was activated by cadaverine, putrescine, and spermine, but not by spermidine or triethylamine (Fig. 2*A*). Putrescine and spermine elicited a half maximal response (EC_50_) at concentrations of 56 μM and 28 μM, respectively, in cells expressing sTAAR365 (Fig. 2*B*). These are comparable to the potency of odorant receptor agonists in similar assays, ranging from 100 nM to 100 μM (25, 26, 27). Surprisingly, sTAAR365 was exquisitely sensitive to cadaverine, with EC_50_ of 4 nM, and a response threshold approaching 100 pM (Fig. 2*B*). This level of sensitivity rivals the olfactory responses observed through *in vivo* recording (17). Moreover, the maximal efficacy (E_max_) of sTAAR365 response to cadaverine was comparable to that for putrescine, whereas spermine elicited a maximal response of only one-third as much, suggesting that spermine likely acts as a partial agonist for sTAAR365.

We confirmed expression of *sTaar365* and *mTaar9* in olfactory sensory neurons (OSNs) with *in situ* hybridization. For adult male and female sea lamprey, the antisense *sTaar365* labeled cells were sparsely distributed in lamellae along the rostral-caudal axis of the main olfactory epithelium, displaying tall cell bodies situated in the deeper epithelium and long dendrites coursing toward the epithelium surface (Fig. S2). In comparison, no labeling was observed with the sense probe. The expression pattern of *sTaar365* is very similar to that of *mTaar9* in the mouse olfactory epithelium (Fig. S3). These results demonstrate that sTAAR365 and mTAAR9 are both broadly tuned and sparsely distributed in olfactory epithelia.

### sTAAR365 and mTAAR9 have convergent and divergent residues for polyamine recognition

A previous study of zTAAR13c by Li *et al.* (13) proposed that Asp^3.32^ and Asp^5.42^ each interact with one of the two amino groups in diamines, such as cadaverine and putrescine. However, it is not known how TAARs recognize polyamines with more than two amino groups. We sought to model the polyamine recognition sites, including residues that directly interact with additional amino groups, in sTAAR365 and mTAAR9. We speculated that TAARs use Asp^3.32^ to confer critical and direct interactions with one amino group, while other nearby polar and/or aromatic residues stabilize polyamine binding by forming hydrogen bond or pi–cation interactions. To test this hypothesis, we generated sTAAR365 and mTAAR9 homology models using GPCR-I-TASSER to predict the putative recognition residues for biogenic polyamines. The models were based on the crystal structures of nine homologous templates. The primary models of sTAAR365 and mTAAR9 shared a maximal identity of 31% and 40%, respectively, to their closest homologous template, the human β_2_-adrenergic GPCR (Protein Data Bank Entry 2rh1A). The homology model with the highest C-score was chosen as the final structure for molecular docking. Both the sTAAR365 and mTAAR9 models displayed a canonical GPCR structure with seven hydrophobic transmembrane α-helices and an eighth intracellular helix (H8) in the C-terminus.

**Figure 3:**
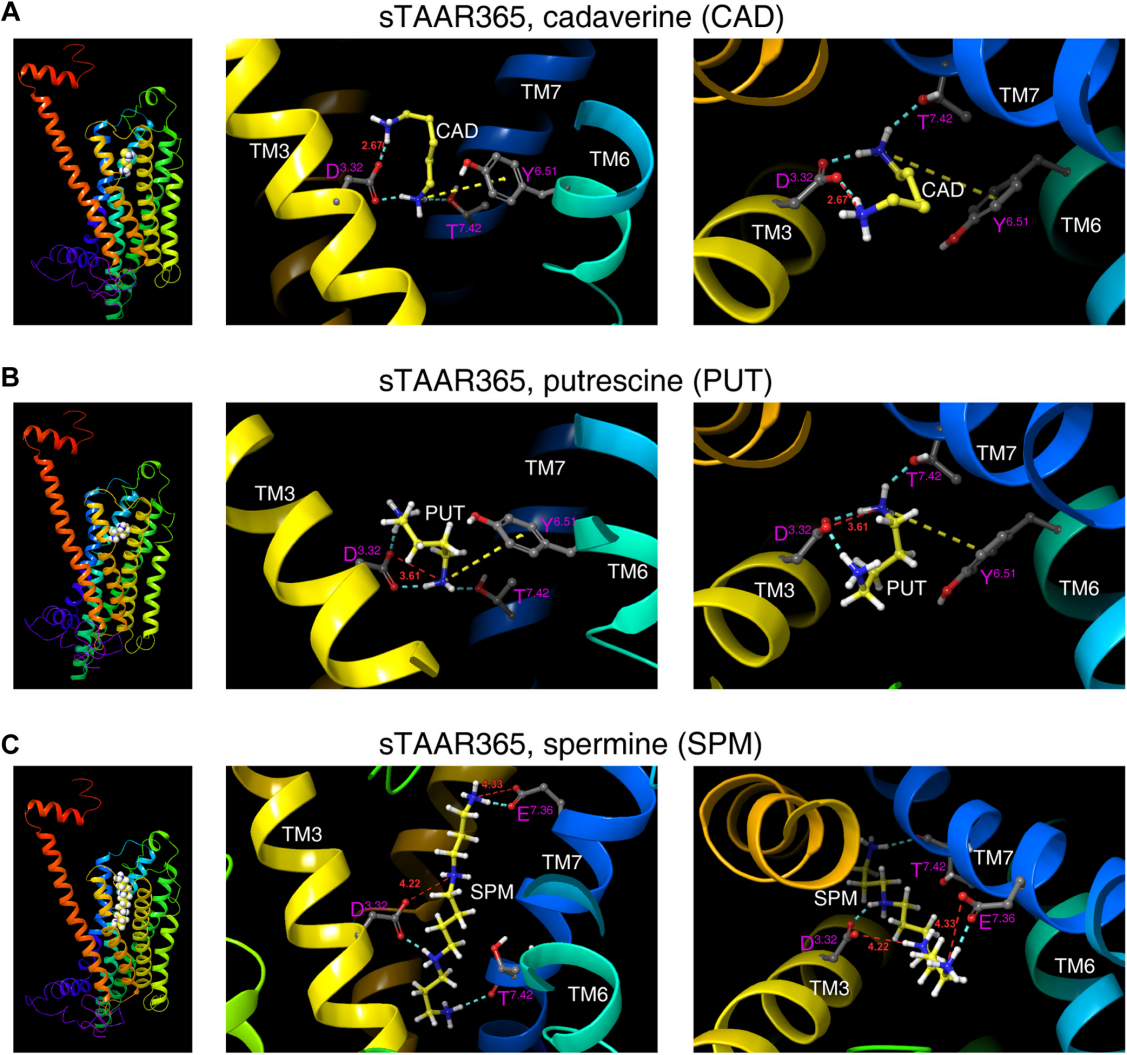
**The binding sites for cadaverine, putrescine, and spermine predicted by docking into sTAAR365 homology model.** Side profile and enlarged views of sTAAR365 homology model predicted spatial position of ligand (*A*) cadaverine (CAD), (*B*) putrescine (PUT), (*C*) spermine (SPM) and corresponding residues involved in ligand recognition. Side chains of key residues involved in ligand binding and major interactions between ligand and predicted binding residues were displayed. *Red dashed line*, salt bridge; *cyan dashed line*, hydrogen bond; *yellow dashed line*, Pi–cation interaction. Distances of the predicted salt bridges in Å were labeled in *red*.

Using the homology models, we performed Induced Fit Docking (IFD) with Schrodinger Maestro 11.5 to predict the residues of sTAAR365 and mTAAR9 involved in polyamine binding. Several poses of ligand–receptor interactions were generated, and the top result was chosen according to docking scores and glide models. For sTAAR365, the highly conserved Asp^3.32^ contacts both amino groups of cadaverine, one is docked 2.67 Å away from the highly conserved Asp^3.32^, forming a salt bridge and a hydrogen bond with the carboxyl group of Asp^3.32^ (Fig. 3*A*). The second amino group of cadaverine also forms a pi–cation interaction with Tyr^6.51^ and a hydrogen bond with Thr^7.42^ (Fig. 3*A*). Similar to cadaverine, putrescine was predicted to interact with Asp^3.32^, Tyr^6.51^, and Thr^7.42^ (Fig. 3*B*). Notably, the distance between the carboxyl group of Asp^3.32^ and its salt-bridged amino group of putrescine is predicted at 3.61 Å. The difference in the salt bridge distance predicted for cadaverine and putrescine likely explains the 1000-fold difference in their potency for sTAAR365.

We then docked spermine, a polyamine with four amino groups, into sTAAR365 homology model to infer how TAARs may interact with additional amino groups. As expected, the cadaverine recognition sites Asp^3.32^ and Thr^7.42^ are involved in spermine binding (Fig. 3*C*). Asp^3.32^ forms a salt bridge and a hydrogen bond with the two middle amino groups of spermine. The distance between the charged aspartate side chain and the further amino group of spermine is 4.22 Å. Meanwhile, an amino group at one end of spermine contacts the backbone of Thr^7.42^ with a hydrogen bond, while the amino group at the other end is anchored on the negatively charged residue Glu^7.36^, located in the extracellular vestibule of TM VII, through a salt bridge and a hydrogen bond. The distance between the carboxyl group of Glu^7.36^ and the terminal amino group of spermine is predicted at 4.33 Å. The salt bridges involved in spermine recognition are longer than the typical cutoff value for a salt bridge at 4 Å. These relatively weak ionic interactions may explain the partial activation of sTAAR365 by spermine.

**Figure 4:**
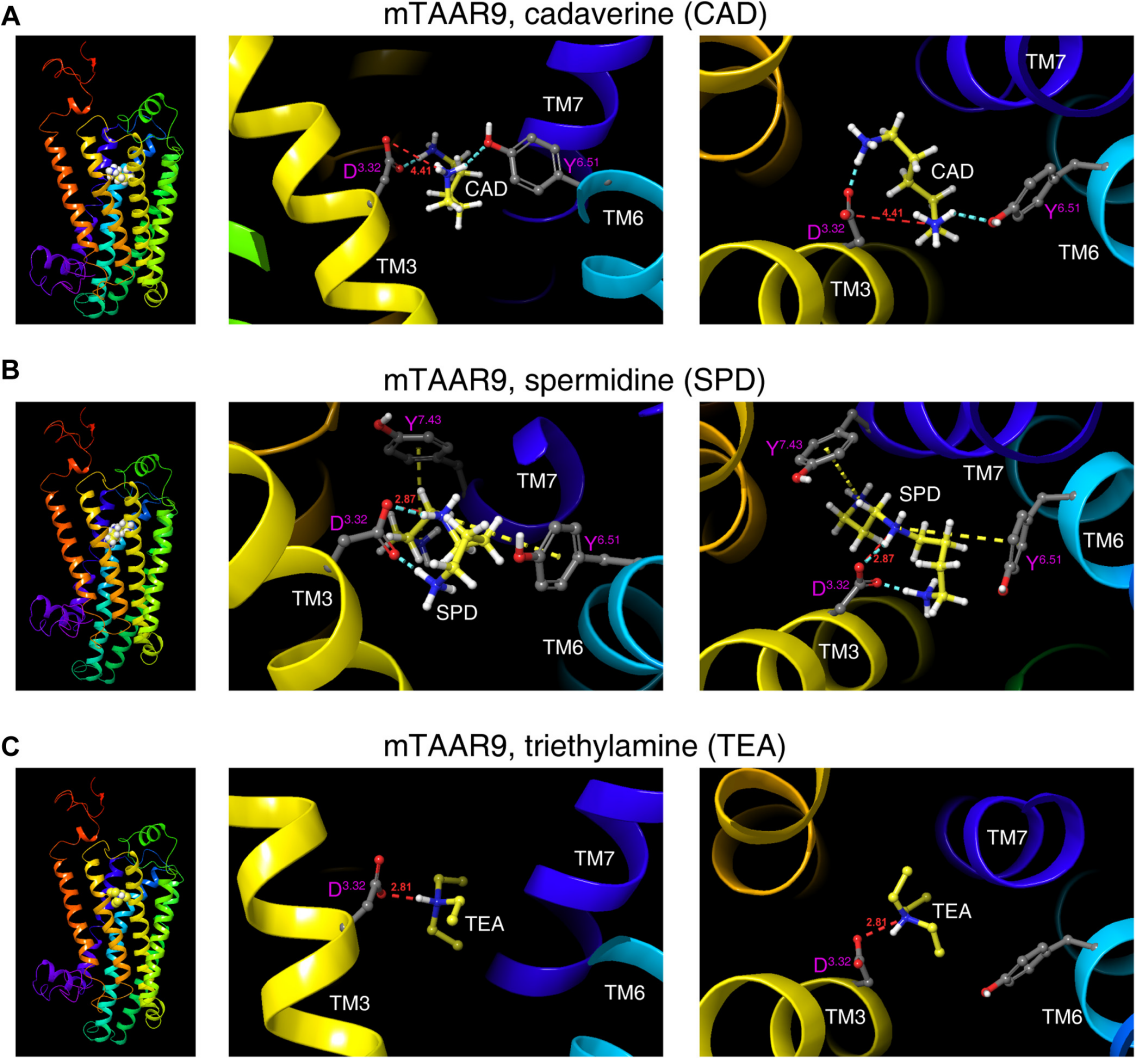
**The binding sites for cadaverine, spermidine, and triethylamine predicted by docking to mTAAR9 homology model.** Side profile and enlarged views of mTAAR9 homology model predicted spatial position of ligand (*A*) cadaverine (CAD), (*B*) spermidine (SPD), (*C*) triethylamine (TEA), and corresponding residues involved in ligand recognition. Side chains of key residues involved in ligand binding and major interactions between ligand and predicted binding residues were displayed. *Red dashed line*, salt bridge; *cyan dashed line*, hydrogen bond; *yellow dashed line*, Pi–cation interaction. Distances of the predicted salt bridges in Å were labeled in *red*.

Docking cadaverine into mTAA9 homology model suggested that Asp^3.32^ and Tyr^6.51^ are the primary binding sites. The carboxyl group of Asp^3.32^ forms a salt bridge with one amino group of cadaverine at a distance of 4.41 Å (Fig. 4*A*) and a hydrogen bond with the second amino group of cadaverine. Likewise, Tyr^6.51^ is predicted to be part of the cadaverine-binding pocket, forming a hydrogen bond with the amino group of cadaverine (Fig. 4*A*). However, mTAAR9 differs from sTAAR365 by having Val^7.42^ instead of Thr^7.42^. The larger distance of the salt bridge and the lack of scaffold interaction with Thr^7.42^ may explain the much lower potency of cadaverine for mTAAR9 compared with sTAAR365. mTAAR9 exhibits robust responses to spermidine, a biosynthetic precursor of spermine. Results from docking spermidine (with three amino groups) into the mTAAR9 model suggested that an extra residue, Tyr^7.43^, cooperates with Asp^3.32^ and Tyr^6.51^ to form the binding pocket (Fig. 4*B*). Asp^3.32^ recognizes the middle amino group of spermidine by a salt bridge, at a distance of 2.87 Å, and a hydrogen bond. Moreover, a pi–cation interaction is also predicted between the Tyr^6.51^ residue and the middle amino group. For the amino groups at the ends of spermidine, Tyr^7.43^ recognizes one and forms a pi–cation interaction, whereas Asp^3.32^ recognizes the amino group at the other end and forms a hydrogen bond (Fig. 4*B*). Furthermore, triethylamine, which has a similar potency as cadaverine for mTAAR9, forms a single salt bridge with the carboxyl group of Asp^3.32^ at a distance of 2.81 Å (Fig. 4*C*).

Taken together, these docking results suggest that sTAAR365 and mTAAR9 have the conserved Asp^3.32^ and Tyr^6.51^ that interact with two amino groups in diamines. In addition, sTAAR365 uses an extra Thr^7.42^ to stabilize recognition of cadaverine and putrescine. The diamine recognition motifs, in addition to an extra Glu^7.36^ in sTAAR365 or Tyr^7.43^ in mTAAR9, enable the selective recognition of spermine and spermidine, respectively.

### The conserved Asp^3.32^ is critical for TAAR activation

**Figure 5:**
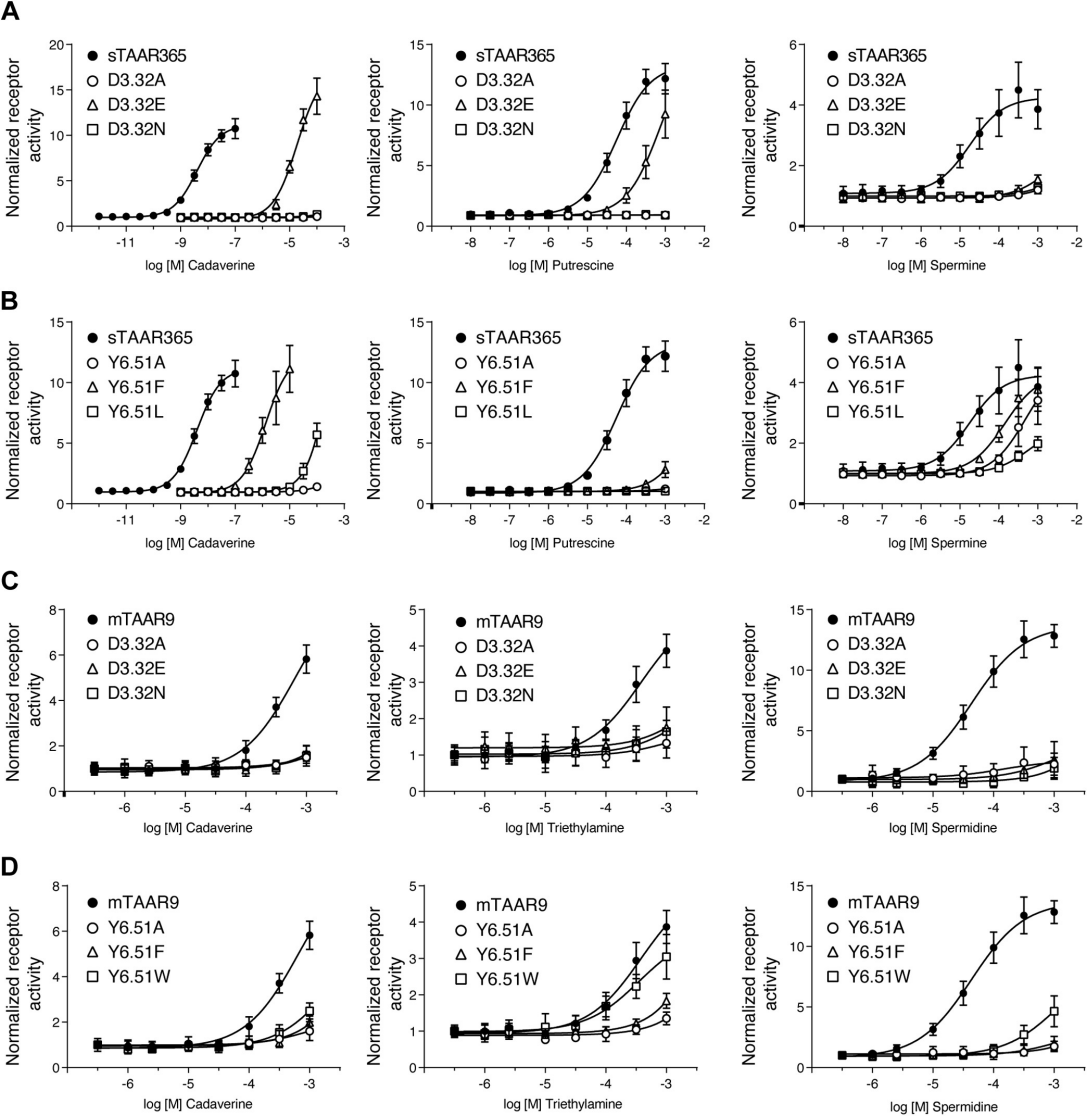
**Effect of amino acid residue substitution at conservative binding sites (Asp**^**3.32**^**and Tyr**^**6.51**^**) of sTAAR365 and mTAAR9.** HEK293T or Hana3A cells were transfected with either wild-type sTAAR365, wild-type mTAAR9, or mutant receptors (Asp^3.32^ or Tyr^6.51^) of sTAAR365 and mTAAR9, and incubated with dilutions of ligands. Receptor activity was normalized to the basal activity of buffer-treated cells (mean ± S.D., n = 3). *A*, concentration-dependent cAMP production of HEK293T cells expressing sTAAR365 and its D^3.32^ mutants stimulated with cadaverine, putrescine, and spermine. *B*, concentration-dependent cAMP production of HEK293T cells expressing sTAAR365 and its Y^6.51^ mutants stimulated with cadaverine, putrescine, and spermine. *C*, concentration-dependent luciferase activity of Hana3A cells expressing mTAAR9 and its D^3.32^ mutants stimulated with cadaverine, spermidine, and triethylamine. *D*, concentration-dependent luciferase activity of Hana3A cells expressing mTAAR9 and its Y^6.51^ mutants stimulated with cadaverine, spermidine, and triethylamine.

To confirm the role of Asp^3.32^ in polyamine recognition by sTAAR365 and mTAAR9, we replaced their Asp^3.32^ with alanine, asparagine, or glutamate. In sTAAR365, a charge-neutralizing mutation of Asp^3.32^ (D3.32A or D3.32N) eliminated activation by cadaverine, putrescine, and spermine (Fig. 5*A*). In contrast, the D3.32E mutant, with the most conservative exchange of Asp^3.32^ to Glu, was activated by cadaverine and putrescine with comparable efficacy as wild-type, albeit with drastically decreased potency (Fig. 5*A* and Table S1). The EC_50_ values of cadaverine and putrescine for the D3.32E mutant increased three and one orders of magnitude for sTAAR365, respectively (Table S1). However, spermine activity at the D3.32E mutant was almost eliminated, resulting in minimal cAMP accumulation at the highest concentration tested (1 mM) (Fig. 5*A*).

Similar results were observed for mutations of Asp^3.32^ in mTAAR9 for responses to spermidine. Activity was abolished for the D3.32A, D3.32N, and D3.32E mutants (Fig. 5*C*). Likewise, cadaverine- and triethylamine-induced activities were abolished for mTAAR9 mutants (Fig. 5*C*). These empirical findings are consistent with docking predictions that the highly conserved Asp^3.32^ is a critical determinant for activation of sTAAR365 and mTAAR9 by various amines.

### Tyr^6.51^ is another common binding partner for polyamine recognition in sTAAR365 and mTAAR9

As described above, the Tyr^6.51^ residue of sTAAR365 was predicted to interact with cadaverine and putrescine. We substituted the Tyr^6.51^ residue with phenylalanine, alanine, or leucine and examined the effect on receptor activity. These substitutions resulted in a loss of the hydrogen-bond interaction or pi-cation, or both. The sTAAR365 Y6.51A and Y6.51L mutants drastically reduced receptor responses to cadaverine and putrescine (Fig. 5*B*). In contrast, cadaverine was able to activate the Y6.51F mutant but with a potency 350-fold lower than the wild-type sTAAR365 (Fig. 5*B* and Table S1). These results strongly suggest that the pi–cation interaction formed by Tyr^6.51^ is important for binding of cadaverine and putrescine to sTAAR365. Though Tyr^6.51^ was not predicted to be directly involved in spermine recognition by sTAAR365, it is in proximity to spermine (within a cutoff value of 4.0 Å) and likely helps stabilize the spermine-binding pocket. Indeed, all three sTAAR365 Tyr^6.51^ mutants were activated by spermine with reductions in potency, ranging from 5- to 20-fold (Fig. 5*B* and Table S1).

To examine whether the Tyr^6.51^ residue of mTAAR9 plays a similar role in polyamine recognition, we substituted Tyr^6.51^ with alanine, phenylalanine, or tryptophan. The Y6.51W mutant showed reduced potency for cadaverine and spermidine, with only small residual activity induced at very high ligand concentrations (Fig. 5*D*). The Y6.51A and Y6.51F mutants almost completely lost the receptor activity to cadaverine and spermidine (Fig. 5*D*). Tyr^6.51^ was not predicted to be a triethylamine recognition site. However, this residue is situated 5 Å from triethylamine in the docking model, well within the range of van der Waals interactions. For triethylamine, Tyr^6.51^ mutants impaired the receptor activity (Fig. 5*D*). In conclusion, results from site-directed mutagenesis suggest that the Tyr^6.51^ residue either constitutes or stabilizes an amine-binding pocket for polyamines in sTAAR365 and mTAAR9.

### Thr^7.42^ is a distinct polyamine recognition motif in sTAAR365

**Figure 6:**
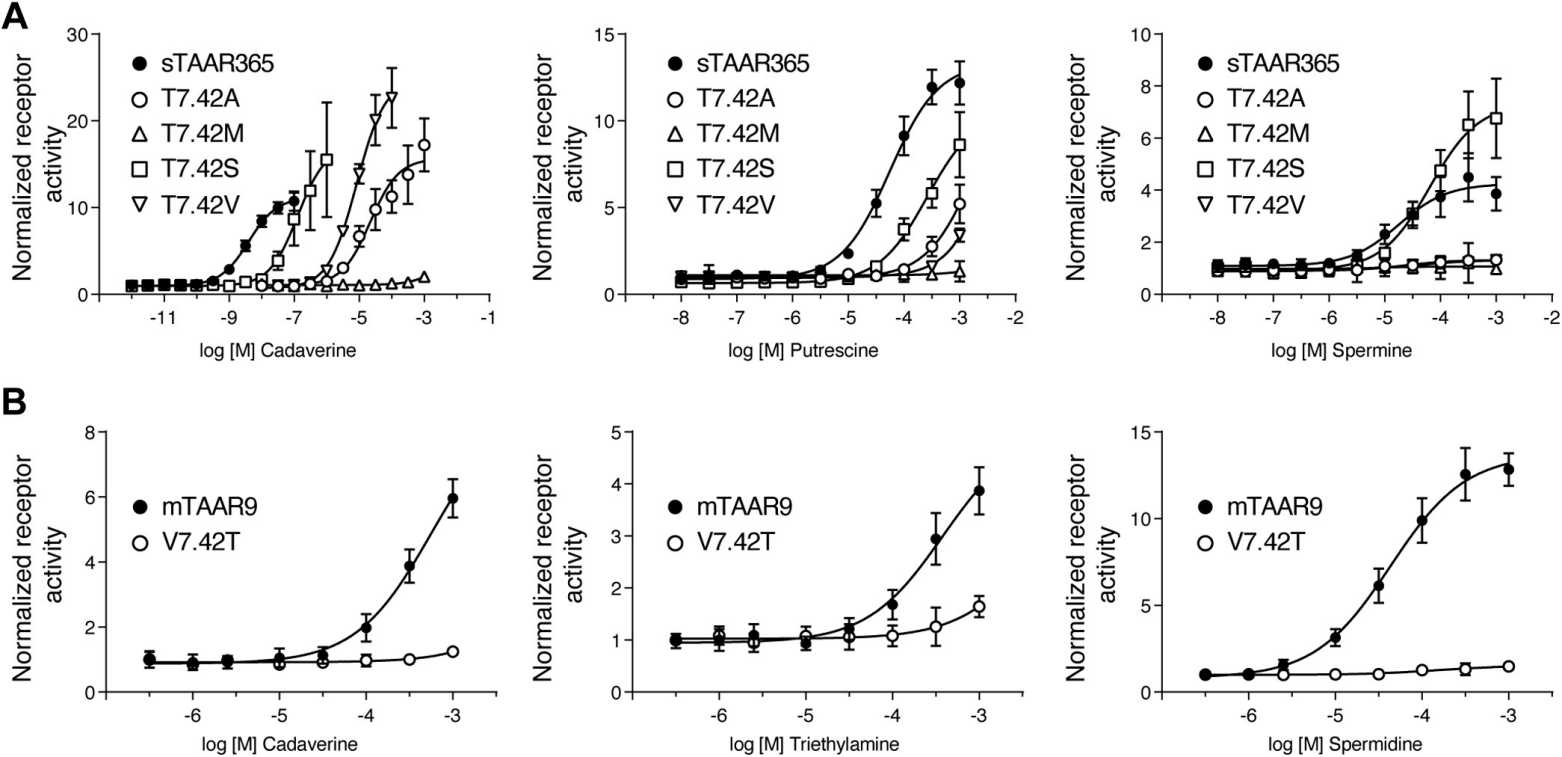
**Concentration–response curves of sTAAR365 and mTAAR9 mutated at a sTAAR365 specific amine recognition site (Thr**^**7.42**^**).** HEK293T or Hana3A cells were transfected with wild-type sTAAR365, wild-type mTAAR9, or Thr^7.42^ mutants of sTAAR365 and mTAAR9, and incubated with dilutions of ligands. Receptor activity was normalized to the basal activity of buffer-treated cells (mean ± S.D., n = 3). *A*, concentration-dependent cAMP production of HEK293T cells expressing sTAAR365 and its T^7.42^ mutants when stimulated with cadaverine, putrescine, and spermine. *B*, concentration-dependent luciferase activity of Hana3A cells expressing mTAAR9 and the V7.42T mutant stimulated with cadaverine, spermidine, and triethylamine.

Our docking studies of sTAAR365 predicted that the polar residue Thr^7.42^ is involved in amine recognition by forming a potential hydrogen bond. We substituted the Thr^7.42^ residue with alanine, valine, methionine, or serine to examine the effect on receptor activity. As the most conservative exchange, T7.42S mutant retained a comparable maximum response to the wild-type when exposed to polyamines (Fig. 6*A*). However, the potency of cadaverine, putrescine, and spermine for the mutant decreased about 32-fold, 5-fold, and 2-fold, respectively (Table S1). In contrast, T7.42A and T7.42V mutants showed drastic decreases in their response to cadaverine, increasing the EC_50_ values by over three orders of magnitude (Fig. 6*A* and Table S1). Likewise, we observed that these two mutants showed a massive decrease in potency of putrescine (Fig. 6*A*). Furthermore, spermine induced no receptor activity in these two mutants (Fig. 6*A*). Finally, the T7.42M mutant, with a bulky side chain that blocks the binding site, lost receptor activity to all ligands tested (Fig. 6*A*).

Since mTAAR9 possesses a hydrophobic Val^7.42^, as opposed to a polar Thr^7.42^ in sTAAR365, we determined whether a V7.42T mutant enhances the potency of polyamines for mTAAR9. Unexpectedly, the mTAAR9 V7.42T mutant lost activity to cadaverine and spermidine and showed a drastic reduction in its response to triethylamine (Fig. 6*B*). Taken together, these results suggest that sTAAR365 utilizes a polar Thr^7.42^ residue to form a hydrogen bond that recognizes polyamines, which is a mechanism distinct from mammalian TAAR9.

### Glu^7.36^ in the extracellular vestibule contributes to spermine recognition in sTAAR365

**Figure 7:**
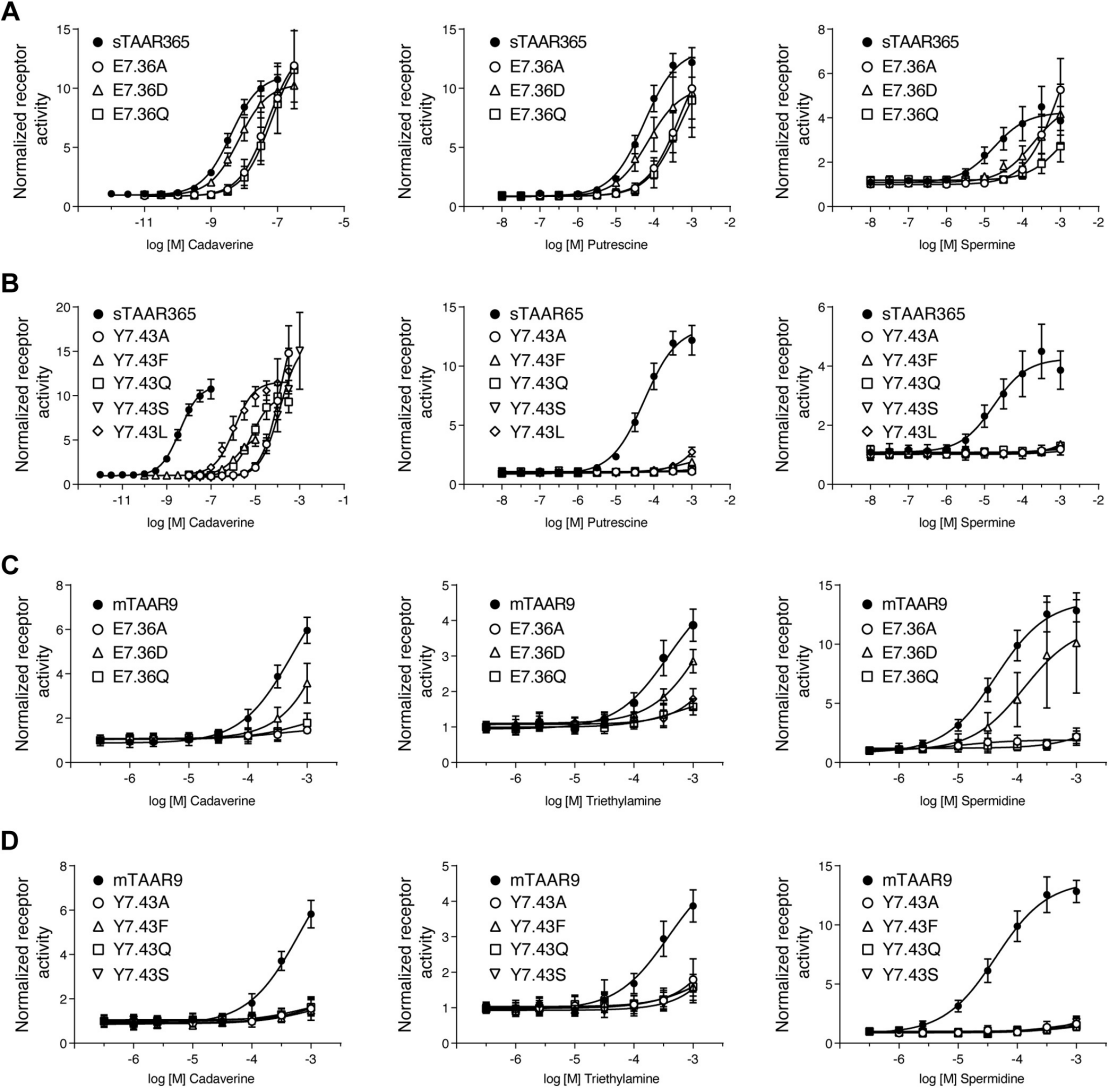
**Altering sTAAR365 and mTAAR9 responses by mutation of divergent binding sites (Glu**^**7.36**^**and Tyr**^**7.43**^**).** HEK293T or Hana3A cells were transfected with wild-type sTAAR365, wild-type mTAAR9, or mutant receptors (Glu^7.36^ and Tyr^7.43^) of sTAAR365 and mTAAR9, and incubated with dilutions of ligands. Receptor activity was normalized to the basal activity of buffer-treated cells (mean ± S.D., n = 3). *A*, concentration-dependent cAMP production of HEK293T cells expressing sTAAR365 and its E^7.36^ mutants stimulated with cadaverine, putrescine, and spermine. *B*, concentration-dependent cAMP production of HEK293T cells expressing sTAAR365 and its Y^7.43^ mutants stimulated with cadaverine, putrescine, and spermine. *C*, concentration-dependent luciferase activity of Hana3A cells expressing mTAAR9 and its E^7.36^ mutants stimulated with cadaverine, spermidine, and triethylamine. *D*, concentration-dependent luciferase activity in Hana3A cells expressing mTAAR9 and its Y^7.43^ mutants stimulated with cadaverine, spermidine, and triethylamine.

Docking of spermine into sTAAR365 homology model suggested that the Glu^7.36^ residue in the extracellular vestibule forms a salt bridge with an amino group at the distal end of spermine. However, Glu^7.36^ was not predicted to interact directly with the docked cadaverine and putrescine. It could also form an interhelical salt bridge with the Arg^2.64^ residue to potentially modulate structural stability of sTAAR365 (Fig. S4). We reasoned that mutations of the Glu^7.36^ residue may drastically reduce the potency of spermine but not cadaverine and putrescine. To test this hypothesis, we generated a series of mutants by replacing the Glu^7.36^ residue with alanine, glutamine, or aspartate. As expected, E7.36A, E7.36Q, and E7.36D had decreased responses to spermine, with increases in EC_50_ values of more than 30-fold (Fig. 7*A* and Table S1). In contrast, the most conservative substitution, the E7.36D mutant, was activated by cadaverine and putrescine with similar EC_50_ values as wild-type sTAAR365 (Fig. 7*A* and Table S1). Similar results were observed in the charge-neutralizing mutants E7.36A and E7.36Q that showed a slight decrease in responses to cadaverine and putrescine, likely due to the loss of the interhelical salt bridge (Fig. 7*A*).

Likewise, the interhelical salt bridge between Glu^7.36^ and Arg^2.64^ was observed in mTAAR9 docking models (Fig. S5). We generated three mutants for the Glu^7.36^ residue (E7.36A, E7.36G, and E7.36D) in mTAAR9 and examined their receptor activity. Triethylamine, cadaverine, and spermidine activated the E7.36D mutant with little loss of potency (Fig. 7*C*). In sharp contrast, the charge-neutralizing mutants E7.36A and E7.36Q completely abolished mTAAR9 activation (Fig. 7*C*). These results indicate that the interhelical salt bridge between Glu^7.36^ and Arg^2.64^ may play an important role in modulating the stability of amine recognition pocket or structural conformation of mTAAR9.

### Tyr^7.43^ either stabilizes or recognizes polyamines

Although the Tyr^7.43^ residue was not predicted to be part of the polyamine recognition motif in sTAAR365 homology model, docking studies suggested that the hydroxyl group of Tyr^7.43^ interacts with Asp^3.32^ by a hydrogen bond that stabilizes the conformation of the essential amine recognition site (Fig. S6). We posited that disruption of the hydrogen bond could drastically reduce the potency of ligands for sTAAR365. To test this hypothesis, we replaced the Tyr^7.43^ residue with alanine, glutamine, phenylalanine, serine, or leucine. As expected, all five sTAAR365 mutants showed decreases in cadaverine potency, up to a 1000-fold (Table S1). In contrast, we observed that all mutants had a reduced maximum response when putrescine was applied (Fig. 7*B*). The Y7.43F and Y7.43L mutants retained minimal putrescine-induced receptor activity, whereas the remaining Tyr^7.43^ mutants completely abolished receptor activity. Likewise, all five mutants were inactive to spermine (Fig. 7*B*). Taken together, all experimental data from the Tyr^7.43^ mutants are consistent with the prediction that Asp^3.32^ is anchored in place by a hydrogen bond to the hydroxyl group of Tyr^7.43^, which stabilizes polyamine recognition of sTAAR365.

A similar interhelical hydrogen bond was not observed in the mTAAR9 homology model; instead, the Tyr^7.43^ residue was predicted to interact with an amino group at the distal end of spermidine through a pi–cation interaction. Functional testing with spermidine revealed that all four mTAA9 mutants of Tyr^7.43^ (Y7.43A, Y7.43Q, Y7.43F, and Y7.43S) eliminated the activity of the receptor (Fig. 7*D*). Furthermore, these mutants did not show receptor responses to cadaverine or triethylamine (Fig. 7*D*). However, flow cytometry analysis showed that mutation of the mTAAR9 Tyr^7.43^ residue to Ala (Y7.43A), Phe (Y7.43F), and Ser (Y7.43S) had impaired cell-surface expression of the receptor (Fig. S10). None of the other tested sTAAR365 mutants exhibited reduced surface expression (Fig. S9). These results suggest that Tyr^7.43^ has an important role in mediating the structural stability of mTAAR9, and mutation of Tyr^7.43^ may result in detrimental conformational alternations. Indeed, we observed that the Tyr^7.43^ residue forms an intramolecular pi–pi stacking interaction with Tyr^7.44^ in docking models of cadaverine and triethylamine (Fig. S7). In all, we speculate that Tyr^7.43^ not only participates in spermidine recognition but also contributes to the structural stability of mTAAR9.

### Trp^7.40^ modulates the function of the aminergic DW motif

**Figure 8:**
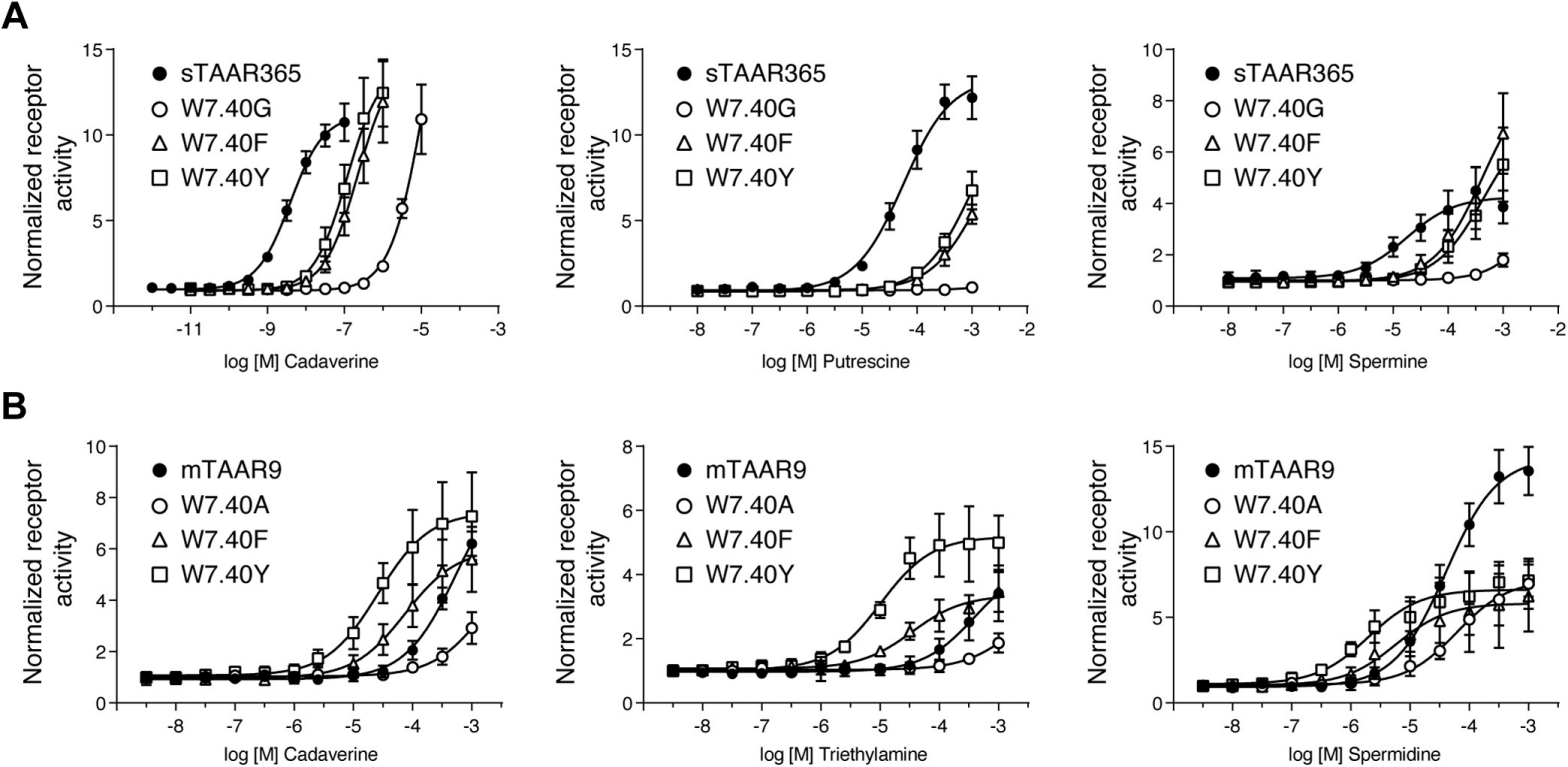
**Altering sTAAR365 and mTAAR9 responses by mutation of Trp**^**7.40**^**in the conserved aminergic DW motif.** HEK293T or Hana3A cells were transfected with wild-type sTAAR365, wild-type mTAAR9, or Trp^7.40^ mutants of sTAAR365 and mTAAR9, and incubated with concentration-dependent ligands. Receptor activity was normalized to the basal activity of buffer-treated cells (mean ± S.D., n = 3). *A*, concentration-dependent cAMP production of HEK293T cells expressing sTAAR365 and its W^7.40^ mutants stimulated with cadaverine, putrescine, and spermine. *B*, concentration-dependent luciferase activity of Hana3A cells expressing mTAAR9 and its W^7.40^ mutants stimulated with cadaverine, spermidine, and triethylamine.

The Trp^7.40^ residue of the aminergic DW motif has been shown to participate in ligand recognition in several aminergic receptors, including serotonin, histamine, muscarinic, adrenergic, and dopamine receptors (23, 28, 29, 30, 31). However, this residue was not included in the polyamine recognition sites of our sTAAR365 or mTAAR9 homology model. To explore whether Trp^7.40^ affects polyamine recognition in sTAAR365 and mTAAR9, we generated a series of mutants (sTAAR365: W7.40G, W7.40F, and W7.40Y; mTAAR9: W7.40A, W7.40F, and W7.40Y) for Trp^7.40^. All three sTAAR365 mutants showed reduction in potency to cadaverine, with increases in EC_50_ values for about 56-fold, 32-fold, and 3000-fold, respectively (Fig. 8*A* and Table S1). For spermine, the EC_50_ for the W7.40F and W7.40Y mutants increased by 20-fold (Fig. 8*A* and Table S1). These two mutants also showed a drastic reduction in their responses to putrescine (Fig. 8*A*). The W7.40G mutant lost receptor activity to putrescine and spermine (Fig. 8*A*). All experimental results suggest that Trp^7.40^ is critical for polyamine recognition by sTAAR365, potentially stabilizing a positively charged amino group *via* hydrophobic or aromatic/aromatic interactions. A close investigation of the docking model for cadaverine and spermine indicates that Trp^7.40^ contacts Tyr^7.43^ with a pi–pi stacking to stabilize Asp^3.32^ in the polyamine-binding pocket (Fig. S8).

**Figure 9:**
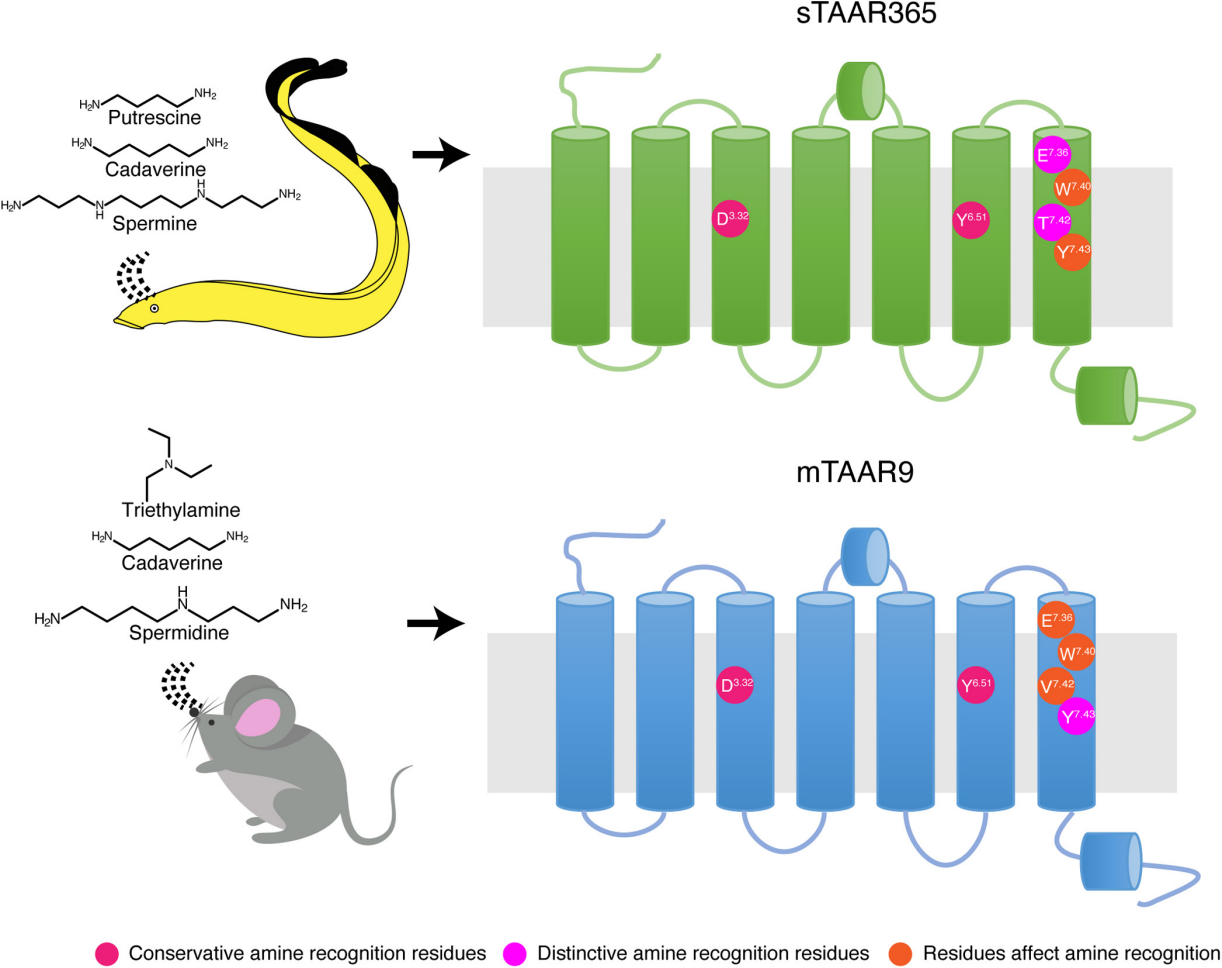
**A schematic diagram describing the proposed polyamine recognition sites in convergent sTAAR365 and mTAAR9.***Cartoon* representations of sTAAR365 (*green*) and mTAAR9 (*blue*) structures show seven transmembrane domains and two short α-helices. Conservative amine recognition residues (Asp^3.32^ and Tyr^6.51^) are indicated with *carnation circle dots*. Distinctive amine recognition residues (Glu^7.36^ and Thr^7.42^ in sTAAR365; Tyr^7.43^ in mTAAR9) are labeled with *magenta circle dots*. *Tangerine circle dots* represent residues (Trp^7.40^ and Tyr^7.43^ in sTAAR365; Glu^7.36^, Trp^7.40^, and Val^7.42^ in mTAAR9) that affect amine recognition.

In sharp contrast to sTAAR365 mutants, the mTAAR9 W7.40F and W7.40Y mutants displayed a marked increase in potency of cadaverine and spermidine, resulting in a shift of the concentration–response curves to lower concentrations by about one order of magnitude (Fig. 8*B*). The efficacy of cadaverine for these two mutants was comparable to wild-type mTAAR9, whereas the efficacy of spermidine was significantly decreased (Fig. 8*B*). For monoamine detection, similar results were observed for these two mutants with triethylamine activation, with an 11-fold and 36-fold decrease of EC_50_, respectively (Fig. 8*B* and Table S1). On the other hand, we observed that the W7.40A mutant showed a decrease in efficacy and potency of cadaverine, spermidine, and triethylamine (Fig. 8*B*). Thus, all experiments performed with the Trp^7.40^ mutants showed that substituting Trp^7.40^ with amino acid residues having a less bulky aromatic side chain increases the potency of ligands at mTAAR9. It is conceivable that the less bulky aromatic residue facilitates access of ligands into the internal binding pocket and generates a more sensitive receptor for amine detection. In contrast, the W7.40A mutation might result in a loss of structural stability by replacing the aromatic side chain with a methyl side chain, which abrogates potential aromatic interactions with neighboring amino acids, or it may reduce receptor overall stability. Taken together, these observations suggest that the Trp^7.40^ residue of the aminergic DW motif plays different pharmacological roles in modulating amine response profiles in sTAAR365 and mTAAR9.

## Discussion

Olfactory TAARs enable vertebrates to detect volatile or soluble amines that are ubiquitous in their habitat. The structural determinants for TAARs that recognize amines with a wide range of amino groups have remained elusive, largely due to the lack of crystal structures for any olfactory receptor. In the present study, we examined the structural basis of sTAAR365 and mTAAR9 for detection of polyamines with up to four amino groups. The sea lamprey and jawed vertebrate TAARs are suggested to have evolved independently, forming distinct clades. Receptors from both clades recognize polyamines, providing a unique opportunity to characterize polyamine recognition sites across independently evolved vertebrate GPCRs. Previous studies have demonstrated that teleost TAARs utilize a canonical amine-detection site Asp^3.32^ or a noncanonical amine-detection site Asp^5.42^ (or both) to recognize various monoamines or diamines (13). However, sTAAR365 and mammalian TAAR9s contain the canonical Asp^3.32^ but lack the Asp^5.42^, implying that these receptors may have evolved alternative mechanisms to recognize various polyamines with two or more amino groups. Based on the odotope theory (25), we propose a hypothetical structural model for polyamine recognition by two clades of independently evolved TAARs, represented by sTAAR365 and mTAAR9. The highly conserved Asp^3.32^ is the primary site essential to binding to one amino group of polyamines. Near Asp^3.32^ are secondary sites that either recognize other amino groups of polyamines (a common residue Tyr^6.51^ for both sTAAR365 and mTAAR9 and a distinct residue Thr^7.42^ for sTAAR365) or stabilize recognition of polyamines with more than two amino groups (Glu^7.36^ and Tyr^7.43^ for both sTAAR365 and mTAAR9). Our models provide strong evidence for a steric and functional odotope theory that sTAAR365 and mTAAR9 utilize convergent structural bases with distinct residues to detect various biogenic polyamines. The convergent mechanism of polyamine recognition by TAARs reveals additional insights into amine detection by GPCRs.

### Conserved and common amine recognition sites for polyamines

The olfactory system uses a combinatorial approach to encode odor identities in which each receptor recognizes multiple odorants, and each odorant activates a specific combination of receptors (25). Interestingly, olfactory systems in different vertebrate species can evolve independently to detect particular odorants, probably through evolutionarily conserved and divergent recognition sites (12). Prior to this study, the Asp^3.32^ residue of the aminergic DW motif (Asp^3.32^ and Trp^7.40^) has been reported as a critical element for amine recognition by TAARs (11, 12, 13, 32). This negatively charged Asp^3.32^ is conserved in all jawless fish TAARs (sea lamprey, 26/26), most mammalian TAARs (mouse, 13/15, rat 15/17, human 6/6), and some teleost TAARs (zebrafish 34/112). A few mammalian TAARs, such as two mouse TAARs (mTAAR7a and mTAAR7d) and two rat TAARs (rTAAR7a and rTAAR7c), contain a noncanonical amine-detection site Glu^3.32^ on transmembrane III. Here, we also showed that Asp^3.32^, but not Trp^7.40^, forms a salt bridge with one amino group in polyamines, which is an essential element for polyamine recognition by both sTAAR365 and mTAAR9. The Asp^3.32^ residue is highly conserved among class A GPCRs, and a similar salt bridge between Asp^3.32^ and the ligand amino group has been described in crystal structures of aminergic receptors, including β1 adrenergic receptor, β2 adrenergic receptor, and H1 histamine receptor (33, 34, 35). Charge-neutralizing mutation of Asp^3.32^ abolished the ability of sTAAR365 and mTAAR9 to detect polyamines. However, mutation of Asp^3.32^ to Glu in sTAAR365 retained receptor activity to polyamines but reduced ligand potency. Thus, the salt bridge formed by the amino group of polyamines and the anionic carboxylate group of residue 3.32 is relatively flexible in sTAAR365, with Asp^3.32^ having a stronger interaction than Glu^3.32^. This phenomenon is consistent with the fact that some mammalian TAARs use Glu^3.32^ to detect amines (11, 13). For mTAAR9, however, the same mutation of Asp^3.32^ to Glu resulted in a nearly complete loss of receptor activity. The differences suggest that other distinct sites in the two receptors are also critical to stabilize polyamine binding.

The polyamine detection mechanisms vary throughout TAAR clades. Previous studies have shown that several zebrafish family members TAAR13 and TAAR14 evolved a noncanonical Asp^5.42^ along with Asp^3.32^ to form a salt bridge with each amino group of dicationic polyamines (11, 13). However, Asp^5.42^ is not present in sTAAR365 and mTAAR9, raising the possibility that alternative sites are involved. We combined homology modeling with mutagenesis experiments to show that sTAAR365 and mTAAR9 use a common polar Tyr^6.51^ residue as another polyamine contact site. Notably, residue 6.51 is also considered as an interaction hotspot for aminergic receptors, by forming pi–cation, hydrogen-bond, or hydrophobic interaction with aminergic ligands (36). Though TAARs are distantly related to aminergic receptors, the recognition function of Tyr^6.51^ for polyamines seems to be well conserved in olfactory TAARs and the phylogenetically related aminergic receptors.

### Distinct amine recognition sites for polyamine detection

Our docking and mutagenesis data indicate that a distinctive polar residue (Thr^7.42^) at the same plane of Asp^3.32^ serves as a binding site for polyamines in sTAAR365. Likewise, a polar residue at this position (Tyr^7.42^) is predicted to be part of the amine recognition pocket in crystal structures of aminergic receptors. Indeed, phylogenetic and evolutionary analyses indicate a close relationship between lamprey TAARs and the aminergic serotonin (5-HT)-4 receptor, supporting the idea that lamprey TAARs and aminergic receptors may use a polar residue at 7.42 that selectively recognizes biogenic amines (37, 38, 39). Mutation of this pivotal Thr^7.42^ binding site to hydrophobic residues resulted in a drastic reduction of ligand potency. In contrast, the subtle exchange for another polar residue (serine) showed minimal loss of potency to polyamines. Moreover, we observed that the maximal efficacy of spermine on the T7.42S mutant differs from that on the wild-type. The greater E_max_ value is likely imparted by the ability of GPCRs coupling to G proteins and the formation of agonist-induced active conformation. These findings encouraged us to examine the role of the hydrophobic Val^7.42^ in mTAAR9. In contrast to the requirement for a polar residue at this position in sTAAR365, swapping Thr^7.42^ for Val^7.42^ in mTAAR9 failed to increase the potency of mTAAR9 ligands and impaired receptor activation, suggesting that the residue at position 7.42 plays disparate roles in sTAAR365 and mTAAR9. Thr^7.42^ is involved in polyamine recognition by sTAAR365. In contrast, mTAAR9 has evolved a hydrophobic Val^7.42^ that does not directly contribute to polyamine recognition but plays an important role in maintaining the structural stability of the receptor, conceivably due to effects of hydrophobic residues that point toward the lipid–protein interface.

In addition, Asp^3.32^ is stabilized by a hydrogen bond to the hydroxyl group of Tyr^7.43^ in sTAAR365 docking models that stabilizes polyamine recognition. The Asp^3.32^ residue of mTAAR7e and mTAAR7f is also reported to anchor Tyr^7.43^ with the same hydrogen bond that stabilizes the ionic interaction between Asp^3.32^ and the ligand amino group (11). A similar hydrogen bond between Asp^3.32^ and Tyr^7.42^ has been described in the crystal structures of various aminergic receptors (36), including muscarinic acetylcholine receptors and α1B adrenergic receptor (30, 40, 41, 42). In most cases, impairment of this hydrogen bond between Asp^3.32^ and Tyr^7.42^ reduces potency of biogenic amines, possibly by destabilizing the interaction of ligand amino group with Asp^3.32^ (36). In accordance with these experimental results on aminergic receptors, the sTAAR365 Tyr^7.43^ mutants had pronounced effects on potency of polyamine ligands, suggesting that the hydrogen bond between Tyr^7.43^ and Asp^3.32^ is important in shaping a stabilized amine-binding pocket in sTAAR365. In contrast, this hydrogen bond was not predicted to be present in the mTAAR9 docking models. In mTAAR9, the Tyr^7.43^ residue is demonstrated to either participate in spermidine recognition or stabilize the recognition pocket of cadaverine and triethylamine. These results suggest that Tyr^7.43^ regulates ligand binding in both sTAAR365 and mTAAR9 albeit with different mechanisms.

Aside from the abovementioned key residues in the upper region of transmembrane domains, an extra negatively charged residue, Glu^7.36^, located in the extracellular vestibule of sTAAR365, is predicted to be part of spermine-binding sites. Notably, Glu^7.36^ contacts Arg^2.64^ with an interhelical salt bridge in the homology modeling of sTAAR365 and mTAAR9. Likewise, a similar hydrogen bond is well maintained in all of the available crystal structures of activated muscarinic acetylcholine receptors (mAChRs), serving as a cryptic pocket for mAChR ligands (43). More interestingly, ligand docking slightly shortens the length of the salt bridge in both sTAAR365 and mTAAR9, suggesting that the interaction between Glu^7.36^ and Arg^2.64^ appears to be essential in stabilizing the opening of the amine recognition pocket. Consistent with this, the E7.36D mutant retains the salt bridge with Arg^2.64^ and shows similar ligand potency in sTAAR365 and mTAAR9 (except spermine for sTAAR365). While the more drastic substitutions, E7.63Q and E7.63A, either reduced potency of cadaverine or putrescine for sTAAR365, or radically diminished the magnitude of activation of mTAAR9. In contrast to cadaverine/putrescine, all three sTAAR365 mutants showed a significant loss of potency to spermine, conceivably because spermine has a much longer carbon chain and Glu^7.36^ serves as an extracellular vestibular site to stabilize the terminal amino group of spermine.

We noticed that compared with the wild-type, some sTAAR365 mutants (such as D3.32E with Hill slope 1.44) show steeper slopes in their concentration–response curves for cadaverine. Overall, three mutant receptors with good responses (E_max_ > 0.5 of WT sTAAR365) gave Hill slopes >1.2. The pharmacological mechanism underlying this phenomenon remains to be examined. This may be due to the much lower potency and attendant artifacts of the high concentrations. However, there could be mutation-induced loss of major interaction, which then requires two separate ligands to contact the multiple sites needed to activate the receptor.

### Polyamines are ecologically relevant odorants

Biogenic amines are potent odorants enriched in biological excretions and act as important social cues that elicit distinct behavioral responses in vertebrates (5, 9, 16, 17, 44). TAARs not only play important roles in mediating aminergic signaling in the nervous system but also serve as olfactory receptors (except TAAR1) to specifically detect these odorous amines. We herein show that sea lamprey olfactory sTAAR365 and mammalian TAAR9 subfamily members are broadly tuned to detect multiple biogenic polyamines. Thus, the ability to detect biogenic polyamines is well conserved among vertebrates, from jawless fish to mammals, highlighting the ecological significance of polyamines. Though the broadly tuned sTAAR365 and narrowly tuned sTAA348 showed no response to spermidine in the heterologous expression system (5), *in vivo* calcium imaging experiments with sea lamprey olfactory sensory neurons revealed that spermine and spermidine can activate separate olfactory sensory neurons at high (10^−5^ M) and low (10^−9^ M) concentrations (45). However, we cannot preclude the involvement of sTAAR365 in detecting spermidine because others have reported that amines can robustly activate TAAR3- and TAAR4-expressing OSNs but fail to activate these receptors in heterologous assays (16, 46). On the other hand, TAAR9 can be activated by urine samples from different species (mouse, rat, human, and other mammalian species) with similar sensitivity (7). However, it is still unclear whether these specific polyamines and TAAR pairs are responsible for the observed instinctive animal behaviors. Nevertheless, these published studies together with our results strongly suggest that polyamines are ecologically relevant odorants for TAARs that mediate vertebrate physiology and behavior. It is worth noting that a previous study has revealed direct activation of G_i_/G_o_ proteins by natural polyamines (47), suggesting that polyamines could potentially elicit physiological responses independent on TAARs.

Taken together, our deorphanization of sTAAR365 provided a unique avenue to examine the structural basis of polyamine recognition by TAARs. Different from the previous studies that identified Asp^3.32^ and Asp^5.42^ for diamine recognition, our results show that sTAAR365 and mTAAR9, two independently evolved vertebrate TAARs, utilize convergent (Asp^3.32^ and Tyr^6.51^) and divergent (Thr^7.42^, Glu^7.36^, and Tyr^7.43^) motifs for recognition of polyamine odorants (Fig. 9). These findings demonstrate a novel molecular mechanism for activation of vertebrate TAARs by polyamines. As sea lamprey is an abundant and destructive invasive species in the Laurentian Great Lakes, future studies are needed to examine the role of sTAAR365 and its ligands in mediating behavior.

## Experimental procedures

### Ethics statement

All procedures involving sea lamprey (*Petromyzon marinus*) were approved by the Michigan State University Institutional Animal Use and Care Committee (03/14-054-00 and 02/17-031-00). Sea lamprey used for *in situ* hybridization was euthanized with 3-aminobenzoic acid ethyl ester (MS222; 100 mg/l; Sigma-Aldrich) followed by dissection of the olfactory organ.

All mouse experiments were approved by the Animal Ethics Committee of Shanghai Jiao Tong University School of Medicine and the Institutional Animal Care and Use Committee (Department of Laboratory Animal Science, Shanghai Jiao Tong University School of Medicine, animal protocol number A-2016-049). Mice used for *in situ* hybridization were euthanized with carbon dioxide followed by dissection of the olfactory epithelium.

### Chemicals

Amine compounds of the highest purity available were purchased from Sigma-Aldrich. All chemicals were dissolved in dimethyl sulfoxide (DMSO; Sigma-Aldrich) at a final concentration of 200 mM and stored at −20 °C.

### Cell lines

HEK293T cells used for all sTAAR365 experiments were maintained at 37 °C with 5% CO_2_ and grown in Dulbecco’s Modified Eagle Medium (DMEM; Hyclone) supplemented with 10% fetal bovine serum (FBS; Gibco) and 1× Antibiotic-Antimycotic (Gibco) Hana3A cells derived from HEK293 used for all mTAAR9 experiments were maintained at 37 °C with 5% CO_2_ and grown in DMEM supplemented with 10% fetal bovine serum and 1× Antibiotic-Antimycotic.

### Cloning of mammalian and sea lamprey TAAR genes

All mammalian TAAR genes were cloned from genomic DNA inserted into a modified pcDNA3.1- (Invitrogen) vector containing a Rho-tag (the first 20 amino acids of bovine rhodopsin) as described previously (Table S2) (6). The open reading frame of sTAAR365 was mined from the sea lamprey genome assembly (Pmarinus_7.0). sTAAR365 was cloned from sea lamprey genomic DNA and inserted into Rho-pCMV modified from pCMV-Tag-2B (Agilent Technologies) by introducing a Rho-tag (the first 21 amino acids of bovine rhodopsin) at the N-terminal replacing the intrinsic Flag-tag (Table S2).

### Site-directed mutagenesis of sTAAR365 and mTAAR9

Site-directed mutations of sTAAR365 and mTAAR9 were introduced following the protocol of Quik Change site-directed mutagenesis kit (Agilent Technologies). In brief, PCR primers were designed by Agilent QuikChange Primer Design (Tables S3 and S4) and PCR reactions were performed using PfuUltra High-Fidelity DNA Polymerase with wild-type sTAAR365 and mTAAR9 plasmids as templates. The methylated parental strands were selectively digested with 1 μl DpnI enzyme, and the DpnI-treated PCR products were transformed into DMT chemically competent cells (Transgen Biotech). All mutants were verified by DNA sequencing. Positive colonies for the desired substitutions were grown in LB broth and the plasmids were isolated using an EndoFree mini plasmid DNA purification kit (Tiangen Biotech).

### Functional assay of mTAAR9 and mutants in the Hana3A heterologous system

Hana3A cells were seeded in poly-D-lysine pre-coated 96-well plates at a density of 1× 10^4^ cells per well with 50 μl DMEM medium with 10% FBS and incubated for 24 h at 37 °C with 5% CO_2_. Cells in each well were transfected with 10 ng CRE-Luc, 10 ng pRL-SV40, 10 ng olfactory mRTPs, and 50 ng mTAAR9 or mutants by Lipofectamine 2000 (Invitrogen), and then incubated for 18 h at 37 °C with 5% CO_2_. Subsequently, the media was aspirated and replaced with 50 μl fresh CD293 media (with 1% glutamine) and incubated for 30 min at 37 °C with 5% CO_2_. Then, cells were stimulated with serial dilutions of compounds (diluted in CD293 media) and incubated for 4 h at 37 °C with 5% CO_2_. The firefly luciferase and renilla luciferase activity was measured with a BioTek microplate reader following manufacturer’s instructions.

### Functional assay of sTAAR365 and mutants in the HEK293T heterologous system

HEK293T cells were maintained at 37 °C with 5% CO_2_ and grown in DMEM supplemented with 10% FBS with 1× Antibiotic-Antimycotic. The cAMP production assay was performed in 384-well plates as described in LANCE Ultra cAMP Kit manual (PerkinElmer) to characterize the cAMP production induced in HEK293T cells expressing sTAAR365 and its mutants. Briefly, HEK293T cells were seeded in a 100 mm dish with 3 × 10^6^ cells in 10 ml DMEM medium with 10% FBS and incubated for 24 h at 37 °C with 5% CO_2_. Cells were then transfected with 5 μg pGL4.29, 1 μg pCI-mRTPs, 1 μg pCI-G_αolf_, and 1 μg sTAAR365 plasmid or sTAAR365 mutants and incubated at 37 °C with 5% CO_2_ for 24 h. Transfected cells were detached with 2 ml Versene (Gibco) and transferred to 384-well plates at 5 μl (2000 cells) per well. Then, 5 μl of the 2× compound serial dilutions was added to each well and incubated for 30 min at room temperature. Afterward, 5 μl 4× Eu-cAMP tracer working solution and 5 μl 4× ULight-anti-cAMP working solution were added to each well and incubated for 1 h at room temperature. Plates were read in the Synergy Neo multimode microplate reader for TR-FRET emissions at 620 nm (as internal reference) and 665 nm (as biological response). The ratio of 665/620 allows normalization for the well-to-well variability and interference due to assay components.

### *In situ* hybridizations of *sTaar365* and *mTaar9*

*sTaar365* anti-sense probes were designed against the 372 bp nucleotide sequences of sTAAR365 open reading frame. Amplified fragments were cloned into the pGEM-T vector (Promega) for sequence verification. Plasmids were linearized using restriction enzyme NcoI (anti-sense probe) or SpeI (sense probe) and used for synthesis of digoxigenin (DIG)-labeled RNA probes with DIG RNA labeling kit (SP6/T7) (Roche). *mTAAR9* anti-sense probe was designed against the entire coding region of mTAAR9 (1044 bp). Amplified fragments were cloned into the TOPO TA cloning vector (Invitrogen) for sequence verification. Plasmids were linearized using restriction enzyme NotI and used for synthesis of digoxigenin-labeled RNA probes with DIG RNA labeling kit (SP6/T7).

*In situ* hybridization was conducted following previously described methods by Chung-Davidson and colleagues. Briefly (48), 20 μm frozen sections of olfactory epithelium were hybridized with RNA probes (3 ng/μl) overnight at 65 °C in the hybridization solution (50% deionized formamide, 1× Denhart's solution, 5% dextran sulfate, 750 mM sodium chloride, 25 mM ethylenediaminetetraacetic acid, 25 mM piperazine-N, N′-bis-2-ethanesulfonic acid, 0.25 mg/ml fish sperm DNA, 0.25 mg/ml poly A acid, and 0.2% sodium dodecyl sulfate). Sections were washed three times (5 min each) in 4× saline-sodium citrate (SSC). For high stringency conditions, sections were washed sequentially in 2× SSC with 0.3% Tween-20 and 0.2 × SSC with 0.3% Tween-20 three times (15 min each) at 68 °C. Sections were washed in 0.1× SSC with 0.3% Tween-20 for 15 min followed by three washes (5 min each) in 0.1 M phosphate buffered saline (PBS) with 0.3% Tween-20 at room temperature. The sections were then incubated with blocking solution (1× PBS, 2 mg/ml bovine serum albumin (BSA), 0.3% Tween-20, and 10% normal sheep serum) for 1 h at room temperature, followed by incubation with alkaline phosphatase-conjugated sheep-anti-digoxigenin Fab fragments (1:1000 diluted in blocking solution; Roche) overnight at 4 °C. Hybridization signals were detected by incubating the sections in nitro blue tetrazolium and 5-bromo-4-chloro-3-indolyl phosphate (NBT/BCIP; Thermo Fisher Scientific) for 2 h at room temperature. sTAAR365 slides were counterstained with nuclear fast red (Vector Laboratories) for 5 min at room temperature. The images were captured with a Zeiss Axioskop2 mot plus microscope with a 20 × or 40 × objective. Control experiments (*sTaar365* and *mTaar9* sense probe) were conducted were conducted simultaneously.

### Homology modeling and ligand docking of sTAAR365 and mTAAR9

Homology models of sTAAR365 and mTAAR9 were generated using GPCR-I-TASSER based on the crystal structure of nine homologous templates (Protein Data Bank Entries: 6oijR, 4amjA, 6kuwA, 5zbh, 3d4s, 2rh1A, 6hlpA, 4ib4, 5uenA) (49). Rank of templates represented the top threading templates selected by GPCR-I-TASSER. The primary models of sTAAR36 and mTAAR9 shared a maximal identity of 31% and 40%, respectively, to their closest homologous template, the human beta 2-adrenergic G protein-coupled receptor (Protein Data Bank Entry 2rh1A). The model with the highest C-score (sTAAR365: 0.19 and mTAAR9: 0.06) was chosen as the final structure.

The homology models of sTAAR365 and mTAAR9 for ligand docking were refined to prepared states by using Protein Preparation Wizard module integrated in Schrodinger Suite (50). Protons were added or eliminated according to physiological pH and restrained minimization was performed. The structures of triethylamine, cadaverine, putrescine, spermidine, and spermine were retrieved from PubChem (https://pubchem.ncbi.nlm.nih.gov) and prepared by LigPrep in Maestro (51) with environment adjusted to a physiological pH of 7.0. We then performed receptor-ligand docking in the Induced-Fit Docking module of Schrodinger (52). The center of binding pocket was set to Asp^3.32^ in both mTAAR9 and sTAAR365. Several poses of ligand–receptor interactions were generated, and the final pose was chosen according to the docking score and glide model.

### Immunocytochemistry assay of sTAAR365 and mutants

HEK293T cells were seeded in collagen I coated 24-well glass bottom plates at a density of 5 × 10^4^ cells per well with 1 ml DMEM medium with 10% FBS and incubated for 24 h at 37 °C with 5% CO_2_. Cells were then transfected with 375 ng pGL4.29, 75 ng pCI-mRTPs, 75 ng pEGFP-N1 (Clontech), and 75 ng sTAAR365 plasmid or sTAAR365 mutants and incubated at 37 °C with 5% CO_2_ for 24 h. The null plasmid, pGL4.29, pCI-mRTPs, and pEGFP-N1 were cotransfected as a negative control. Subsequently, 100 μl of 37% formaldehyde was added to each well and incubated for 15 min at room temperature and then washed with 500 μl of PBS three times (5 min each). Cells were permeabilized or not permeabilized with 0.5% Triton X-100 for 10 min at room temperature to label the whole cell or only cell membrane, respectively. Then, cells were washed with 500 μl of PBS three times (5 min each). Permeabilized cells were treated with 500 μl blocking buffer (5% BSA and 0.3% Triton X-100 in PBS) for 1 h at room temperature. Nonpermeabilized cells were treated with 500 μl blocking buffer (5% BSA in PBS) for 1 h at room temperature. Then, the blocking buffer was removed and 300 μl mouse monoclonal anti-rhodopsin antibody (MABN15, Millipore; 1:500 diluted in dilution buffer: 1% BSA in PBS for nonpermeabilized cells or 1% BSA, 0.3% Triton X-100 in PBS for permeabilized cells) was added to each well and incubated at 4 °C overnight. The antibody solution was aspirated and washed five times for 5 min each with 500 μl of PBS. Cells were incubated with 300 μl red-fluorescent Alexa Fluor 594 goat anti-mouse IgG (Invitrogen; 1:500 diluted in dilution buffer: 1% BSA in PBS for nonpermeabilized cells or 1% BSA, 0.3% Triton X-100 in PBS for permeabilized cells) for 1 h at room temperature. Cells were washed three times for 5 min each with 500 μl of PBS. Cells were counterstained with 300 μl DAPI (Invitrogen; 1:5000 diluted in PBS) and incubated in the dark for 5 min. Cells were washed three times for 5 min each with 500 μl of PBS. Images were acquired at 400 × magnification under DMI8 Thunder (Leica) with DAPI filter, GFP filter, and Texas Red filter (ten random views/well, n = 3). The mean value of the red fluorescent signal was quantified with LAS X software (Leica).

### Fluorescence cytometry analysis

Hana3A or HEK293T cells were seeded in 6-well plates at a density of 3 × 10^5^ cells per well with 2 ml DMEM medium with 10% FBS and incubated for 24 h at 37 °C with 5% CO_2_. Cells were then transfected with 0.4 μg pCI-mRTPs, 0.3 μg pEGFP-N1, and 2 μg wild-type receptor plasmid (mTAAR9 or sTAAR365) or mutant receptor plasmid, and incubated at 37 °C with 5% CO_2_ for 24 h. The null pCI plasmid, pCI-mRTPs, and pEGFP-N1 were cotransfected as a negative control. Subsequently, transfected cells were dissociated with CellstripperTM (Corning) and transferred into 5 ml tubes for antibody incubation. 100 μl mouse monoclonal anti-rhodopsin antibody (MABN15, Millipore; 1:100 diluted in staining buffer: 5% BSA, 1% NaN_3_ in PBS) was added to each tube and incubated at 4 °C for 45 min. Cells were washed twice by adding 2 ml staining buffer and centrifuged at 200 × *g* for 3 min at 4 °C. In the next step, 100 μl phycoerythrin-conjugated donkey anti-mouse IgG (Jackson ImmunoResearch; 1:100 diluted in staining buffer: 5% BSA, 1% NaN_3_ in PBS) was added and incubated at 4 °C for 30 min. Cells were washed twice by adding 2 ml staining buffer and then centrifuged at 200 × *g* for 3 min at 4 °C. Cell pellets were resuspended in 500 μl staining buffer for flow cytometry analysis (BD LSRFortessaTM X-20, Becton, Dickinson and Company).

## Availability of data and material

The data that support the findings of this study are available from the corresponding author upon reasonable request. All other data related to receptor expression and activity are contained in the article and supplementary material.

## Supporting information

This article contains supporting information.

## Conflict of interest

The authors declare that they have no conflicts of interest with the contents of this article.
