# Supplementary material for: Convergent olfactory trace amine-associated receptors detect biogenic polyamines with distinct motifs via a conserved binding site
Source: J Biol Chem. 2021 Sep 30;297(5):101268. doi: 10.1016/j.jbc.2021.101268 (PMC8546428; doi:10.1016/j.jbc.2021.101268)
Supplement: Supplemental Figures S1–S10 and Tables S1–S4 [file mmc1.pdf]

Supplementary information

Convergent olfactory trace amine-associated receptors detect biogenic polyamines with distinct motifs via a conserved binding site

Running Title: Polyamine binding sites in convergent olfactory TAARs

Authors: Liang Jia[1,2,3,4]‡, Shengju Li[1,2]‡, Wenxuan Dai[1,2], Lingna Guo[1,2], Zhengrong Xu[1,2,6], Anne M. Scott[3], Zhe Zhang[3], Jianfeng Ren[4], Qinghua Zhang[4], Thomas S. Dexheimer[8], Yu-Wen Chung-Davidson[3], Richard R. Neubig[5*], Qian Li[1,2,7*], Weiming Li[3*]

Correspondence to: liweim@msu.edu; liqian@shsmu.edu.cn; rneubig@msu.edu

This PDF file includes:

Table S1. Detailed EC_{50} information of polyamines for sTAAR365, mTAAR9, sTAAR365 mutants, and mTAAR9 mutants.

Table S2. Primers of PCR for mouse olfactory TAARs, mammalian TAAR9 and sTAAR365.

Table S3. Primers of PCR for mTAAR9 mutants.

Table S4. Primers of PCR for sTAAR365 mutants.

Supplementary Figures S1. Comparison of primary structures of mammalian TAAR9 with sea lamprey sTAAR365 and sTAAR348.

Supplementary Figures S2. Representative OSNs expressing *sTaar365* in the main olfactory epithelium of adult sea lamprey.

Supplementary Figures S3. Representative OSNs expressing *mTaar9* in the mouse olfactory epithelium.

Supplementary Figures S4. Interhelical interactions between $Glu^{7.36}$ and $Arg^{2.64}$ predicted in the homology models of wildtype sTAAR365 and its $Glu^{7.36}$ mutants.

Supplementary Figures S5. Interhelical interactions between $Glu^{7.36}$ and $Arg^{2.64}$ predicted in the homology models of wildtype mTAAR9 and its $Glu^{7.36}$ mutants.

Supplementary Figures S6. $Asp^{3.32}$ is stabilized by an interhelical hydrogen-bond to the hydroxyl group of $Tyr^{7.43}$ in the sTAAR365 homology modeling.

Supplementary Figures S7. $Tyr^{7.43}$ is stabilized by an intramolecular pi-pi stacking to the aromatic side chain of $Tyr^{7.44}$ in mTAAR9 docking models.

Supplementary Figures S8. $Trp^{7.40}$ contacts $Tyr^{7.43}$ with an intramolecular pi-pi stacking to stabilize $Asp^{3.32}$ in sTAAR365 docking models.

Supplementary Figures S9. sTAAR365 mutants are well-expressed on the surface of HEK293T cells with comparable expression level to wildtype sTAAR365.

Supplementary Figures S10. Flow cytometry analysis of mTAAR9 and its mutants.

Table S1. EC_{50} values of cadaverine, putrescine, spermidine, spermine, and triethylamine for wildtype sTAAR365, wildtype mTAAR9, sTAAR365 mutants, and mTAAR9 mutants.

Wildtype/Mutants	EC_{50} (mean ± S.D., n = 3)			Wildtype/Mutants	EC_{50} (mean ± S.D., n = 3)		
	Spermine	Cadaverine	Putrescine		Spermidine	Cadaverine	Triethylamine
WT sTAAR365	28 ± 6 μM	4.0 ± 0.6 nM	56 ± 7 μM	WT mTAAR9	43 ± 7 μM	311 ± 109 μM	251 ± 82 μM
sTAAR365 D3.32A	loss	loss	loss	mTAAR9 D3.32A	loss	loss	loss
sTAAR365 D3.32E	loss	14 ± 0.5 μM	>500 μM	mTAAR9 D3.32E	loss	loss	loss
sTAAR365 D3.32N	loss	loss	loss	mTAAR9 D3.32N	loss	loss	loss
sTAAR365 Y6.51A	319 ± 112 μM	loss	loss	mTAAR9 Y6.51A	loss	loss	loss
sTAAR365 Y6.51F	114 ± 20 μM	1.7 ± 0.9 μM	>500 μM	mTAAR9 Y6.51F	loss	loss	loss
sTAAR365 Y6.51L	>500 μM	>100 μM	loss	mTAAR9 Y6.51W	>1000 μM	>1000 μM	355 ± 80 μM
sTAAR365 T7.42A	loss	31 ± 24 μM	>500 μM	mTAAR9 V7.42T	loss	loss	loss
sTAAR365 T7.42M	loss	loss	loss	mTAAR9 E7.36A	loss	loss	loss
sTAAR365 T7.42S	64 ± 7 μM	126 ± 13 nM	260 ± 129 μM	mTAAR9 E7.36D	142 ± 43 μM	>1000 μM	>1000μM
sTAAR365 T7.42V	loss	9 ± 2 μM	>500 μM	mTAAR9 E7.36Q	loss	loss	loss
sTAAR365 E7.36A	>500 μM	50 ± 20 nM	420 ± 87 μM	mTAAR9 Y7.43A	loss	loss	loss
sTAAR365 E7.36D	>500 μM	7 ± 2 nM	72 ± 14 μM	mTAAR9 Y7.43F	loss	loss	loss
sTAAR365 E7.36Q	>500 μM	60 ± 11 nM	325 ± 169 μM	mTAAR9 Y7.43Q	loss	loss	loss
sTAAR365 Y7.43A	loss	144 ± 11 μM	loss	mTAAR9 Y7.43S	loss	loss	loss
sTAAR365 Y7.43F	loss	2 ± 0.2 μM	>1000 μM	mTAAR9 W7.40F	9 ± 7 μM	83 ± 35 μM	36 ± 16 μM
sTAAR365 Y7.43Q	loss	7 ± 0.3 μM	loss	mTAAR9 W7.40Y	2 ± 0.5 μM	28 ± 8 μM	11 ± 3 μM
sTAAR365 Y7.43S	loss	124 ± 9 μM	loss	mTAAR9 W7.40A	68 ± 48 μM	>1000 μM	>1000 μM
sTAAR365 Y7.43L	loss	1 ± 0.2 μM	>1000 μM				
sTAAR365 W7.40F	564 ± 371 μM	223 ± 37 nM	>500 μM				
sTAAR365 W7.40Y	530 ± 259 μM	127 ± 41 nM	>500 μM				
sTAAR365 W7.40G	loss	12 ± 4 μM	loss				

Table S2. Primers of PCR for mouse olfactory TAARs, mammalian TAAR9 and sTAAR365.

	**Sequence (5'-3')**
**mTAAR2-F**	TTGCGGCCGCGATGGCATCTTTTGAAGCCCAG
**mTAAR2-R**	CGGAATTCCTATTCTGTTTCTTTTTGAGT
**mTAAR3-F**	TTGCGGCCGCGATGGATCTAATATACATTCCC
**mTAAR3-R**	CGGAATTCTTAATGTGCTTCAGGAAAAAG
**mTAAR4-F**	TTGCGGCCGCGATGAATACACCCGACCCCTGG
**mTAAR4-R**	CGGAATTCCTAAGGATGTGCAGGATGCAG
**mTAAR5-F**	TTGCGGCCGCGATGAGAGCTGTCCTCCTCCCGG
**mTAAR5-R**	CGGAATTCCTCAGTCATGGTATAAATCAACA
**mTAAR6-F**	TTGCGGCCGCGATGGGCAGTAACTCGTCTCCG
**mTAAR6-R**	CGGAATTCTTATATTTGCTCAGAGAACAAG
**mTAAR7a-F**	TTGCGGCCGCGATGGACAAATTGGTTGACCATT
**mTAAR7a-R**	CCCTCGAGCTACTCAGGAAACAAGTTGGTG
**mTAAR7b-F**	TTGCGGCCGCGATGGCTACAGATAATGACAGTT
**mTAAR7b-R**	CCCTCGAGCTACTCAGGAAACAAGTTGGTG
**mTAAR7d-F**	TTGCGGCCGCGATGGCTACAGGTGATGACAGTT
**mTAAR7d-R**	CCCTCGAGCTATTCAGGAAACAGGTTGGTG
**mTAAR7e-F**	TTGCGGCCGCGATGGCTACAGGTGATGACAGT
**mTAAR7e-R**	CGGAATTCCTACTCAGGAAACAGGTTGGT
**mTAAR7f-F**	TTGCGGCCGCGATGTCTATAGCTGATGAAACTG
**mTAAR7f-R**	CGGAATTCCTACTCAGAAAACAGATTGGT
**mTAAR8a-F**	TTGCGGCCGCGATGACCAGCAACTTTTCCCAA
**mTAAR8a-R**	CGGAATTCTTACTCTGAAAATAAATTTGCA
**mTAAR8b-F**	TTGCGGCCGCGATGACCAGCAACTTTTCCCAA
**mTAAR8b-R**	CGGAATTCTTACTCTGAAAACAAACTCATG
**mTAAR8c-F**	TTGCGGCCGCGATGACCAGCAACTTTTCCCAA
**mTAAR8c-R**	CGGAATTCTTACTCTGAAAATAAATTTGT
**mTAAR9-F**	TTGCGGCCGCGATGACAAGCGACTTCTCCCCA
**mTAAR9-R**	CGGAATTCTTAACCTGCACCTGCCTCTTC
**human TAAR9-F**	TTGCGGCCGCGATGGTGAACAATTTCTCCCA
**human TAAR9-R**	CGGAATTCTTAATCTGTCTCTACTTCTT

**hamster TAAR9-F**	TTGCGGCCGCGATGGCAAGCAACTTCTCCCCT
**hamster TAAR9-R**	CGGAATTCTTAACCTACATCTGCCTCTTC
**rat TAAR9-F**	TTGCGGCCGCGATGGAGCTCTGCTACGAGAAC
**rat TAAR9-R**	CGGAATTCTTAACCTGCACCTGCTTCTT
**cat TAAR9-F**	TTGCGGCCGCGATGGTGAACAATTCCGCCCA
**cat TAAR9-R**	CGGAATTCTCACTCTATATCTGCTTCCT
**rabbit TAAR9-F**	TTGCGGCCGCGATGGCAGCTGTGGAGTTCTG
**rabbit TAAR9-R**	CGGAATTCTTAATCTATATCTGCCTCTTC
**sTAAR365-F**	TTGCGGCCGCGATGGAAACACTGAACGAGTCC
**sTAAR365-R**	CGGAATTCTCACCTATCTGACTCCTTGCC

Table S3. Primers of PCR for mTAAR9 mutants.

	**Sequence (5'-3')**
**mTAAR9 D3.32A-F**	TTCCACACGTGTTTCGCCACCTCCTTCTGT
**mTAAR9 D3.32A-R**	GCGAAACACGTGTGGAACTTACAGTAACTC
**mTAAR9 D3.32E-F**	TTCCACACGTGTTTCGAAACCTCCTTCTGT
**mTAAR9 D3.32E-R**	TTCGAAACACGTGTGGAACTTACAGTAACT
**mTAAR9 D3.32N-F**	TTCCACACGTGTTTCCAAACCTCCTTCTGT
**mTAAR9 D3.32N-R**	TTGGAAACACGTGTGGAACTTACAGTAACT
**mTAAR9 Y6.51A-F**	CGTGTCCTGGCTGCCAGCCATTATTGATGC
**mTAAR9 Y6.51A-R**	GCTGGCAGCCAGGACACGAGAAATGCAGCC
**mTAAR9 Y6.51F-F**	CGTGTCCTGGCTGCCATTCATTATTGATGC
**mTAAR9 Y6.51F-R**	AATGGCAGCCAGGACACGAGAAATGCAGCC
**mTAAR9 Y6.51W-F**	CGTGTCCTGGCTGCCATGGATTATTGATGC
**mTAAR9 Y6.51W-R**	CCATGGCAGCCAGGACACGAGAAATGCAGC
**mTAAR9 V7.42T-F**	ATTAGTGTGGTGTACTTACTACAATTCAG
**mTAAR9 V7.42T-R**	AGTACACCACACTAATATCTCATAGACGT
**mTAAR9 E7.36A-F**	TGCGTACGTCTATGCGATATTAGTGTG
**mTAAR9 E7.36A-R**	CGCATAGACGTACGCAGGAGTTATGAAG
**mTAAR9 E7.36D-F**	TGCGTACGTCTATGACATATTAGTGTG
**mTAAR9 E7.36D-R**	GTCATAGACGTACGCAGGAGTTATGAAG
**mTAAR9 E7.36Q-F**	CCTGCGTACGTCTATCAGATATTAGTGTGG
**mTAAR9 E7.36Q-R**	GATAGACGTACGCAGGAGTTATGAAGTTCA
**mTAAR9 Y7.43A-F**	ATATTAGTGTGGTGTGTTGCCTACAATTCAGCTATGAACC
**mTAAR9 Y7.43A-R**	GGTTCATAGCTGAATTGTAGGCAACACACCACACTAATAT
**mTAAR9 Y7.43F-F**	TAGTGTGGTGTGTTTTCTACAATTCAGCTA
**mTAAR9 Y7.43F-R**	AAAACACACCACACTAATATCTCATAGACG
**mTAAR9 Y7.43Q-F**	AGTGTGGTGTGTTCAATACAATTCAGCTA
**mTAAR9 Y7.43Q-R**	TTGAACACACCACACTAATATCTCATAGA
**mTAAR9 Y7.43S-F**	AGTGTGGTGTGTTTCCTACAATTCAGCTA
**mTAAR9 Y7.43S-R**	GGAAACACACCACACTAATATCTCATAGA
**mTAAR9 W7.40A-F**	GTCTATGAGATATTAGTGGCGTGTGTTTACTACAATTCAG
**mTAAR9 W7.40A-R**	CTGAATTGTAGTAAACACACGCCACTAATATCTCATAGAC

**mTAAR9 W7.40F-F**	GTCTATGAGATATTAGTGTTCTGTGTTTACTACAATTCAG
**mTAAR9 W7.40F-R**	CTGAATTGTAGTAAACACAGAACACTAATATCTCATAGAC
**mTAAR9 W7.40Y-F**	GTCTATGAGATATTAGTGTATTGTGTTTACTACAATTCAG
**mTAAR9 W7.40Y-R**	CTGAATTGTAGTAAACACAATACACTAATATCTCATAGAC

Table S4. Primers of PCR for sTAAR365 mutants.

	Sequence (5'-3')
sTAAR365 D3.32A-F	GAGTGCAAAGTGTGTAGGCGAACCACGAGTGTATC
sTAAR365 D3.32A-R	GATACACTCGTGGTTCGCCTACACACTTTGCACTC
sTAAR365 D3.32E-F	AGAGTGCAAAGTGTGTACTCGAACCACGAGTGTAT
sTAAR365 D3.32E-R	ATACACTCGTGGTTCGAGTACACACTTTGCACTCT
sTAAR365 D3.32N-F	GAGTGCAAAGTGTGTAGTTGAACCACGAGTGTATCTT
sTAAR365 D3.32N-R	AAGATACACTCGTGGTTCAACTACACACTTTGCACTC
sTAAR365 Y6.51A-F	GTCGACACCACGAAGGCCGGCATCCAGAGCGA
sTAAR365 Y6.51A-R	TCGCTCTGGATGCCGGCCTTCGTGGTGTCGAC
sTAAR365 Y6.51F-F	TCGACACCACGAAGAACGGCATCCAGAGC
sTAAR365 Y6.51F-R	GCTCTGGATGCCGTTCTTCGTGGTGTCGA
sTAAR365 Y6.51L-F	GTTGTCGACACCACGAATAACGGCATCCAGAGCGAG
sTAAR365 Y6.51L-R	CTCGCTCTGGATGCCGTTATTCGTGGTGTCGACAAC
sTAAR365 T7.42A-F	CCGTTGAGTTGATGTACGCGAACCAGTTGATGAACTC
sTAAR365 T7.42A-R	GAGTTCATCAACTGGTTCGCGTACATCAACTCAACGG
sTAAR365 T7.42M-F	CACCGTTGAGTTGATGTACATGAACCAGTTGATGAACTC
sTAAR365 T7.42M-R	GAGTTCATCAACTGGTTCATGTACATCAACTCAACGGTG
sTAAR365 T7.42S-F	CCGTTGAGTTGATGTACGAGAACCAGTTGATGAACTC
sTAAR365 T7.42S-R	GAGTTCATCAACTGGTTCTCGTACATCAACTCAACGG
sTAAR365 T7.42V-F	CACCGTTGAGTTGATGTACACGAACCAGTTGATGAACTCC
sTAAR365 T7.42V-R	GGAGTTCATCAACTGGTTCGTGTACATCAACTCAACGGTG
sTAAR365 E7.36A-F	AACCAGTTGATGAACGCCCACGCCAACGAGC
sTAAR365 E7.36A-R	GCTCGTTGGCGTGGGCGTTCATCAACTGGTT
sTAAR365 E7.36D-F	TGAACCAGTTGATGAAATCCCACGCCAACGAGC
sTAAR365 E7.36D-R	GCTCGTTGGCGTGGGATTTCATCAACTGGTTCA
sTAAR365 E7.36Q-F	CCAGTTGATGAACTGCCACGCCAACGAGC
sTAAR365 E7.36Q-R	GCTCGTTGGCGTGGCAGTTCATCAACTGG
sTAAR365 Y7.43A-F	GATTCACCGTTGAGTTGATGGCCGTGAACCAGTTGATGAACT
sTAAR365 Y7.43A-R	AGTTCATCAACTGGTTCACGGCCATCAACTCAACGGTGAATC
sTAAR365 Y7.43F-F	TCACCGTTGAGTTGATGAACGTGAACCAGTTGATG
sTAAR365 Y7.43F-R	CATCAACTGGTTCACGTTCATCAACTCAACGGTGA

**sTAAR365 Y7.43Q-F**	GATTCACCGTTGAGTTGATCTGCGTGAACCAGTTGATGAAC
**sTAAR365 Y7.43Q-R**	GTTCATCAACTGGTTCACGCAGATCAACTCAACGGTGAATC
**sTAAR365 Y7.43S-F**	GATTCACCGTTGAGTTGATGCTCGTGAACCAGTTGATGAACT
**sTAAR365 Y7.43S-R**	AGTTCATCAACTGGTTCACGAGCATCAACTCAACGGTGAATC
**sTAAR365 Y7.43L-F**	GGATTCACCGTTGAGTTGATTAACGTGAACCAGTTGATGAAC
**sTAAR365 Y7.43L-R**	GTTCATCAACTGGTTCACGTTAATCAACTCAACGGTGAATCC
**sTAAR365 W7.40G-F**	GTTGATGTACGTGAACCCGTTGATGAACTCCCACG
**sTAAR365 W7.40G-R**	CGTGGGAGTTCATCAACGGGTTCACGTACATCAAC
**sTAAR365 W7.40F-F**	TTGAGTTGATGTACGTGAAGAAGTTGATGAACTCCCACGC
**sTAAR365 W7.40F-R**	GCGTGGGAGTTCATCAACTTCTTCACGTACATCAACTCAA
**sTAAR365 W7.40Y-F**	GGCGTGGGAGTTCATCAACTATTTCACGTACATCAACTCAAC
**sTAAR365 W7.40Y-R**	GTTGAGTTGATGTACGTGAAATAGTTGATGAACTCCCACGCC

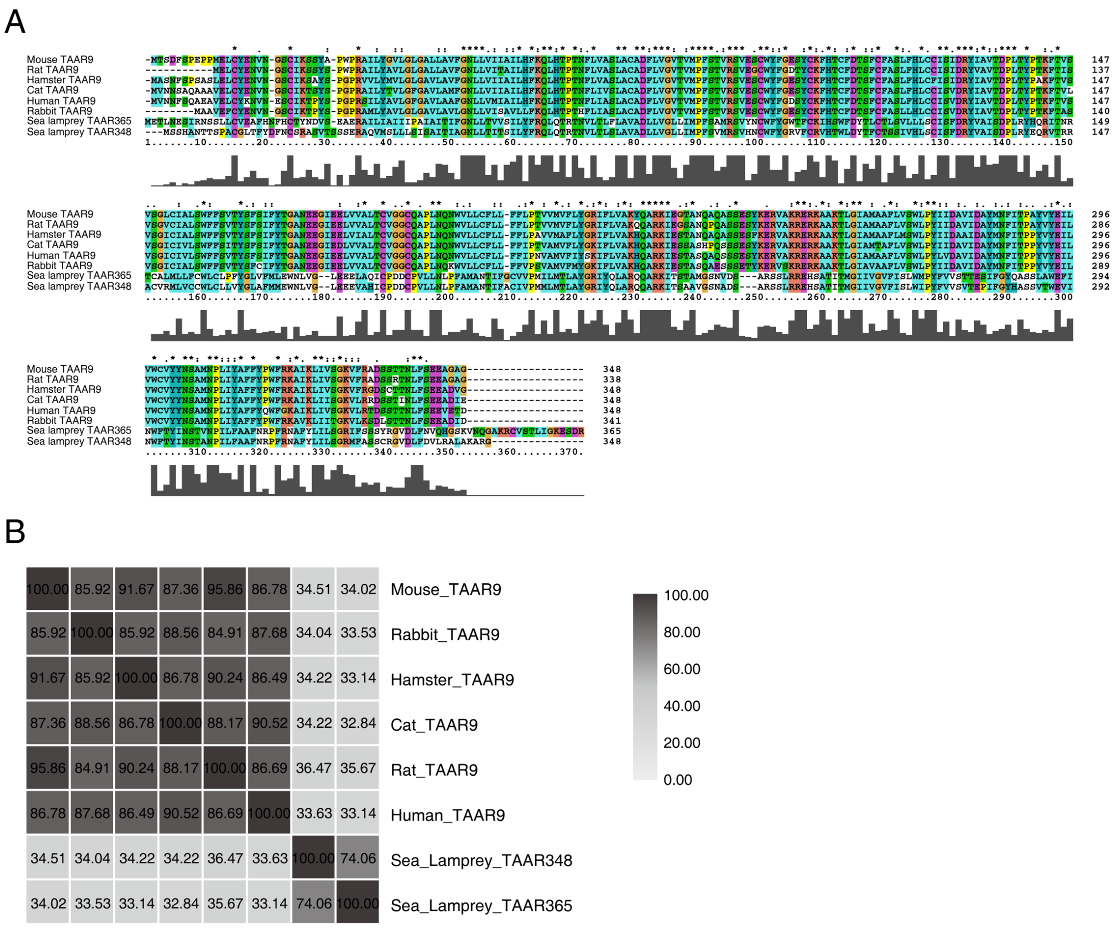

Supplementary Fig S1. Comparison of primary structures of mammalian TAAR9 with sea lamprey sTAAR365 and sTAAR348.

(A) Mouse TAAR9 (NP_001010831.1, 348 amino acid residues), rat TAAR9 (NP_783192.1, 338 amino acid residues), hamster TAAR9 (XP_005088204.1, 348 amino acid residues), cat (XP_003986636.1, 348 amino acid residues), human TAAR9 (NP_778227.3, 348 amino acid residues), and rabbit (XP_002714783.1, 341 amino acid residues) were aligned with sea lamprey sTAAR365 (365 amino acid residues) and sTAAR348 (348 amino acid residues) by using CLUSTAL 2.1 with default parameters. We used positive scores from the Gonnet Pam250 matrix to define strong (score > 0.5) and weak groups (score ≤ 0.5). Color assignment was based on the amino acid residue profile. **(B)** The percentage of identical amino acid residues among mammalian TAAR9 receptors, sea lamprey sTAAR365, and sTAAR348 was calculated using NCBI blast2seq (a pairwise sequence alignment tool, https://blast.ncbi.nlm.nih.gov/Blast.cgi) and plotted.

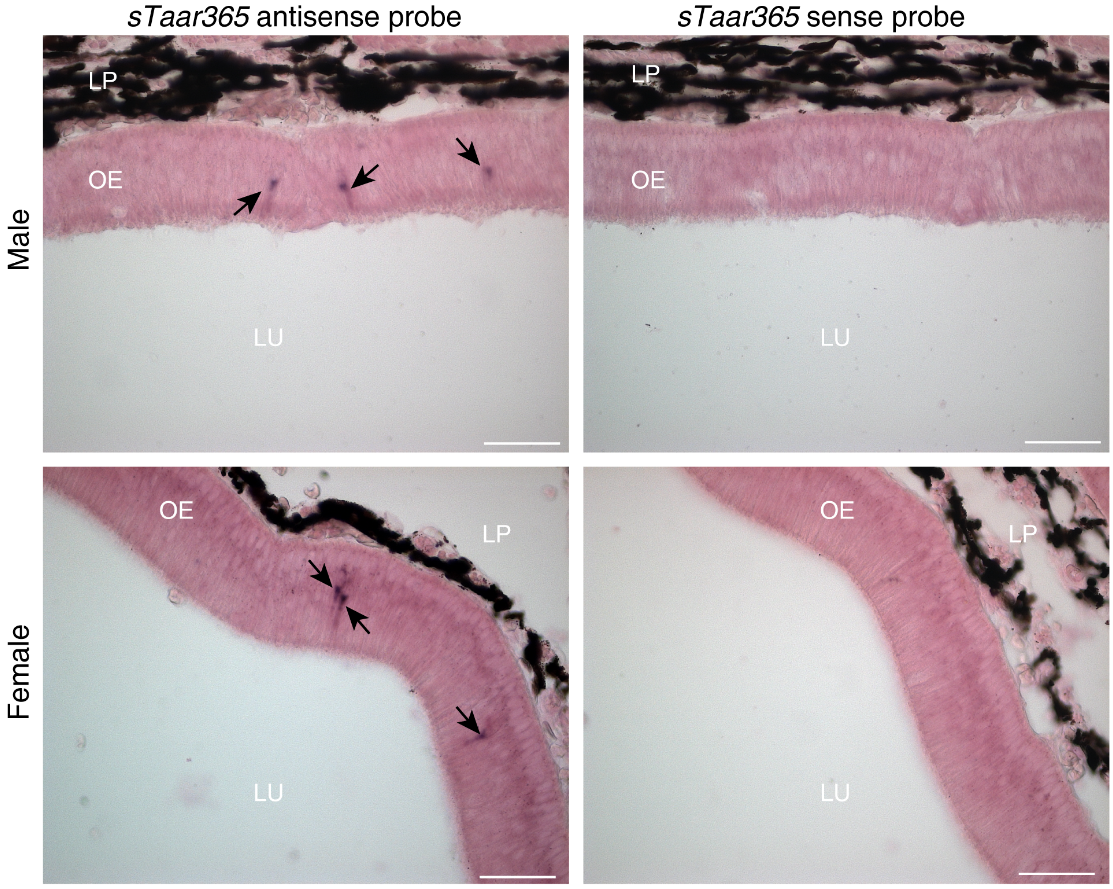

Supplementary Fig S2. Representative olfactory receptor neurons expressing *sTaar365* in a cross-sectional view of the main olfactory epithelium of adult sea lamprey.

Representative olfactory receptor neurons expressing *sTaar365* (purple), labeled with a DIG-labeled antisense RNA probe in a cross-sectional view of the main olfactory epithelium of an adult male and female sea lamprey. Sections were counterstained with Nuclear Fast Red. Positive neurons expressing *sTaar365* indicated with purple stain (NBT/BCIP) are marked by black arrows. No positive neurons were observed in the cross sections with digoxigenin-labeled *sTaar365* sense RNA probe. Black melanophores in the lamina propria are characteristic of sea lamprey olfactory epithelia. Scale bars: 50 µm. LP, lamina propria; LU, lumen; OE, olfactory epithelium.

mTaar9 antisense probe

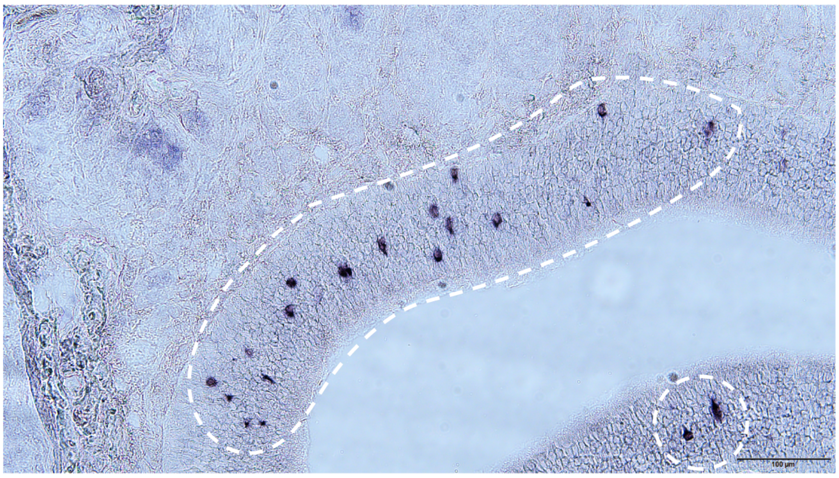

Supplementary Fig S3. Representative olfactory receptor neurons expressing *mTaar9* in a coronal-sectional view of mouse olfactory epithelium.

Representative olfactory receptor neurons expressing *mTaar9* (purple), labeled with a DIG-labeled antisense RNA probe in a coronal-sectional view of mouse olfactory epithelium. Positive neurons expressing *mTaar9* indicated with purple stain (NBT/BCIP) are circled with white dashed lines. Scale bar: 100 µm.

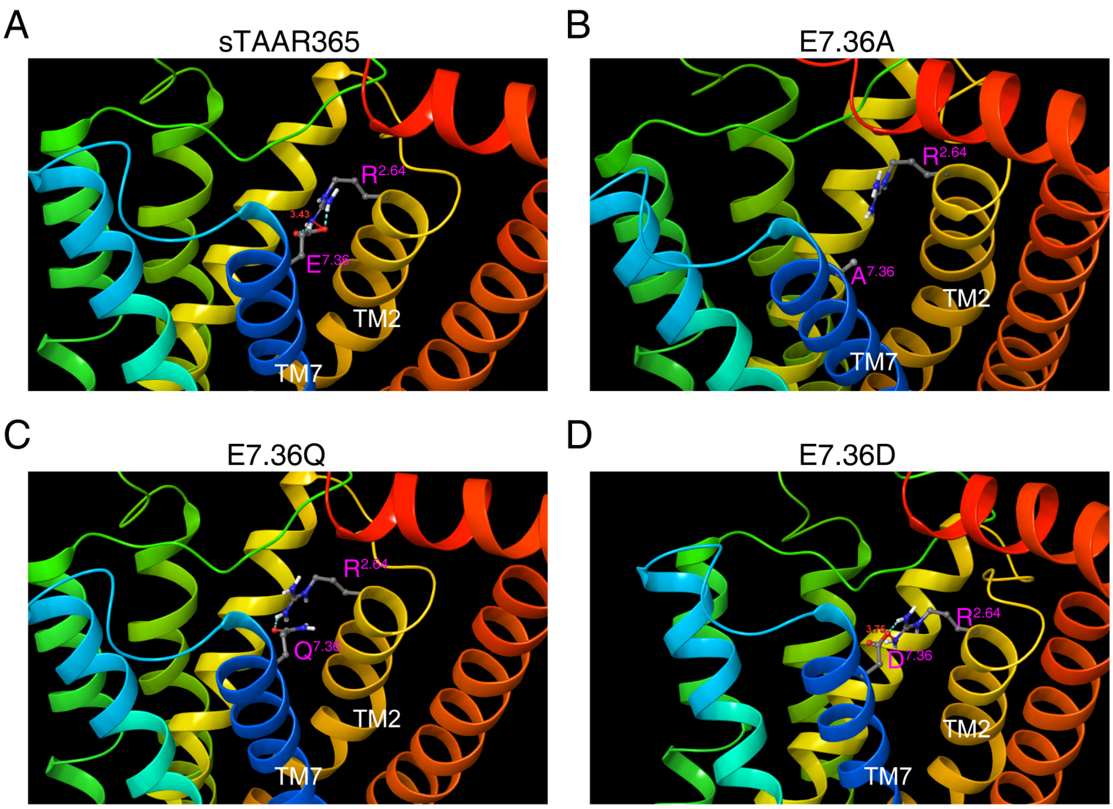

Supplementary Fig S4. Interhelical interactions between Glu$^{7.36}$ and Arg$^{2.64}$ predicted in the homology models of sTAAR365 and its Glu$^{7.36}$ mutants.

Side chains of Glu$^{7.36}$ and Arg$^{2.64}$ and major interactions between these two residues were displayed. Red dashed line, salt bridge; cyan dashed line, hydrogen-bond. Distances of the predicted salt bridges in Å were labeled in red. **(A)** Enlarged view of sTAAR365 predicted interhelical interactions between Glu$^{7.36}$ and Arg$^{2.64}$. **(B)** Enlarged view of mutant E7.36A predicted spatial position of Arg$^{2.64}$ and mutated Ala$^{7.36}$. **(C)** Enlarged view of mutant E7.36Q predicted interhelical interactions between Arg$^{2.64}$ and mutated Gln$^{7.36}$. **(D)** Enlarged view of mutant E7.36D predicted interhelical interactions between Arg$^{2.64}$ and mutated Asp$^{7.36}$.

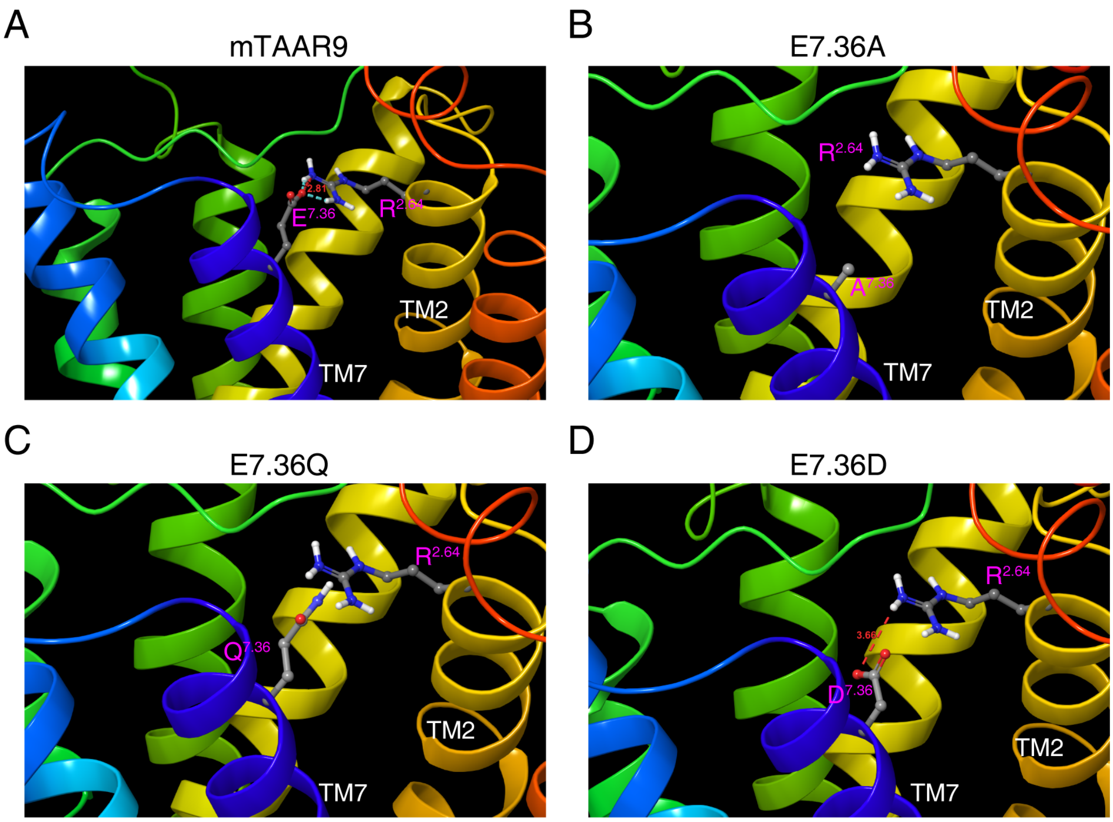

Supplementary Fig S5. Interhelical interactions between $Glu^{7.36}$ and $Arg^{2.64}$ predicted in the homology models of mTAAR9 and its $Glu^{7.36}$ mutants.

Side chains of $Glu^{7.36}$ and $Arg^{2.64}$ and major interactions between these two residues were displayed. Red dashed line, salt bridge; cyan dashed line, hydrogen-bond. Distances of the predicted salt bridges in Å were labeled in red. **(A)** Enlarged view of mTAAR9 predicted interhelical interactions between $Glu^{7.36}$ and $Arg^{2.64}$. **(B)** Enlarged view of mutant E7.36A predicted spatial position of $Arg^{2.64}$ and mutated $Ala^{7.36}$. **(C)** Enlarged view of mutant E7.36Q predicted spatial position of $Arg^{2.64}$ and mutated $Gln^{7.36}$. **(D)** Enlarged view of mutant E7.36D predicted interhelical interactions between $Arg^{2.64}$ and mutated $Asp^{7.36}$.

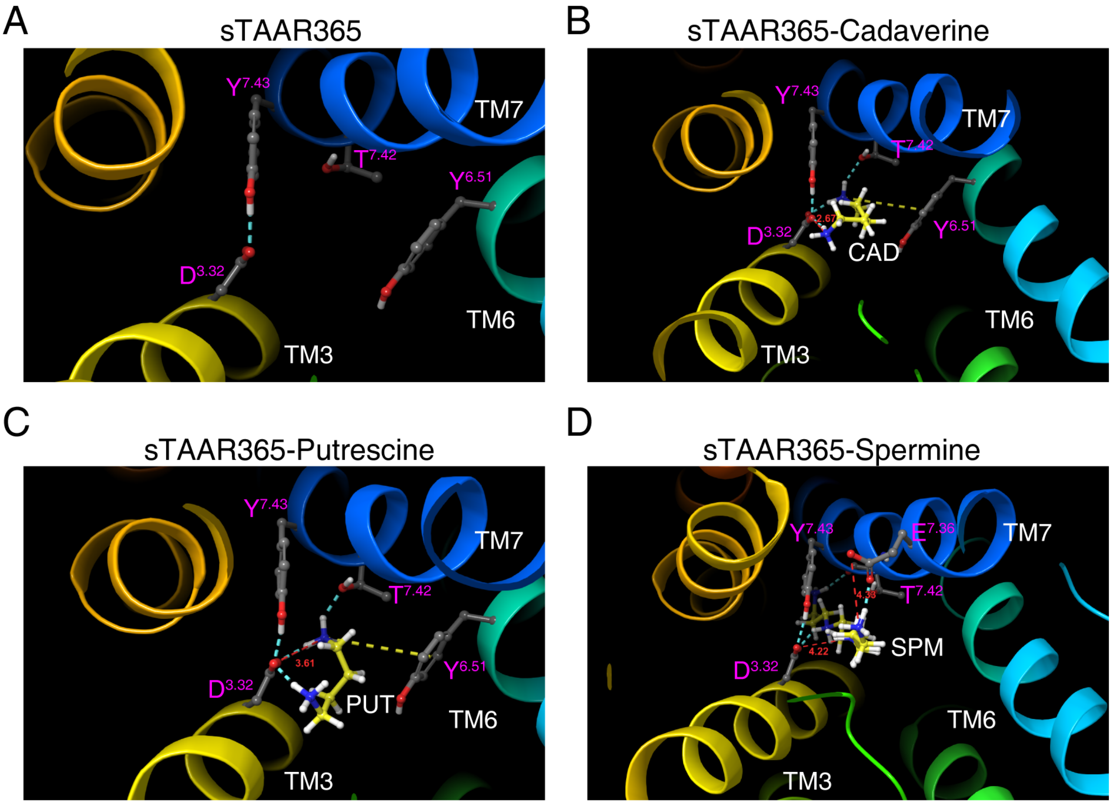

Supplementary Fig S6. Asp$^{3.32}$ is stabilized by an interhelical hydrogen-bond to the hydroxyl group of Tyr$^{7.43}$ in the sTAAR365 homology modeling.

Side chains of key residues, ligand, and major interactions were displayed. Red dashed line, salt bridge; cyan dashed line, hydrogen-bond; yellow dashed line, Pi-cation interaction. Distances of the predicted salt bridges in Å were labeled in red. **(A)** sTAAR365 homology modeling predicted an interhelical hydrogen-bond between Asp$^{3.32}$ and Tyr$^{7.43}$. **(B)** Docking of cadaverine into sTAAR365 homology model predicted an interhelical hydrogen-bond between Asp$^{3.32}$ and Tyr$^{7.43}$. **(C)** Docking of putrescine into sTAAR365 homology model predicted an interhelical hydrogen-bond between Asp$^{3.32}$ and Tyr$^{7.43}$. **(D)** Docking of spermine into sTAAR365 homology model predicted an interhelical hydrogen-bond between Asp$^{3.32}$ and Tyr$^{7.43}$.

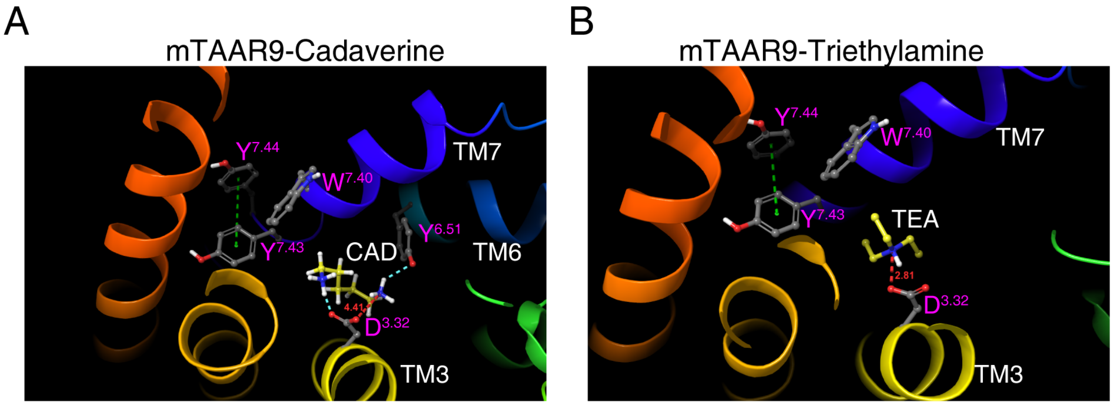

Supplementary Fig S7. Tyr$^{7.43}$ is stabilized by an intramolecular pi-pi stacking to the aromatic side chain of Tyr$^{7.44}$ in mTAAR9 docking models.

Side chains of key residues, ligand, and major interactions were displayed. Red dashed line, salt bridge; cyan dashed line, hydrogen-bond; green dashed line, Pi-pi stacking interaction. Distances of the predicted salt bridges in Å were labeled in red. **(A)** Docking of cadaverine into mTAA9 homology model predicted an intramolecular pi-pi stacking interaction between Tyr$^{7.43}$ and Tyr$^{7.44}$. **(B)** Docking of triethylamine into mTAA9 homology model predicted an intramolecular pi-pi stacking interaction between Tyr$^{7.43}$ and Tyr$^{7.44}$.

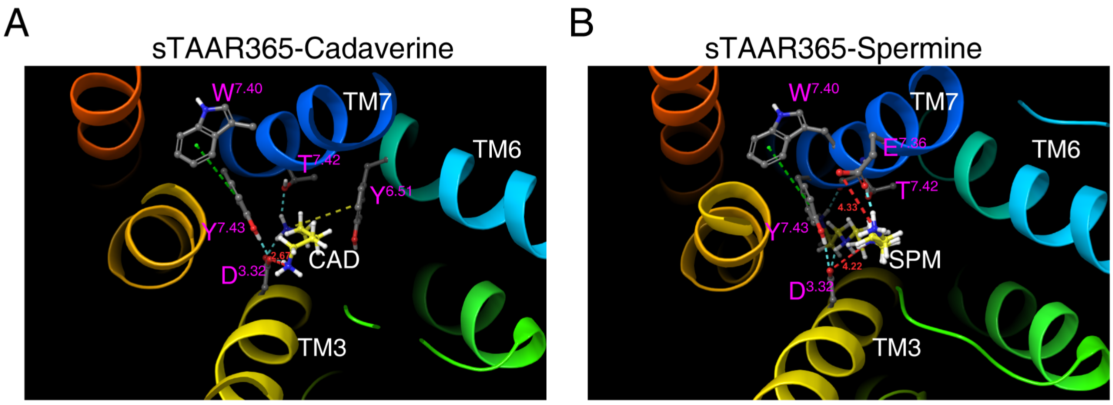

Supplementary Fig S8. Trp$^{7.40}$ contacts Tyr$^{7.43}$ with an intramolecular pi-pi stacking to stabilize Asp$^{3.32}$ in sTAAR365 docking models.

Side chains of key residues, ligand, and major interactions were displayed. Red dashed line, salt bridge; cyan dashed line, hydrogen-bond; green dashed line, Pi-pi stacking interaction; yellow dashed line, Pi-cation interaction. Distances of the predicted salt bridges in Å were labeled in red. **(A)** Docking of cadaverine into sTAAR365 homology model predicted an intramolecular pi-pi stacking interaction between Trp$^{7.40}$ and Tyr$^{7.43}$. **(B)** Docking of spermine into sTAAR365 homology model predicted an intramolecular pi-pi stacking interaction between Trp$^{7.40}$ and Tyr$^{7.43}$.

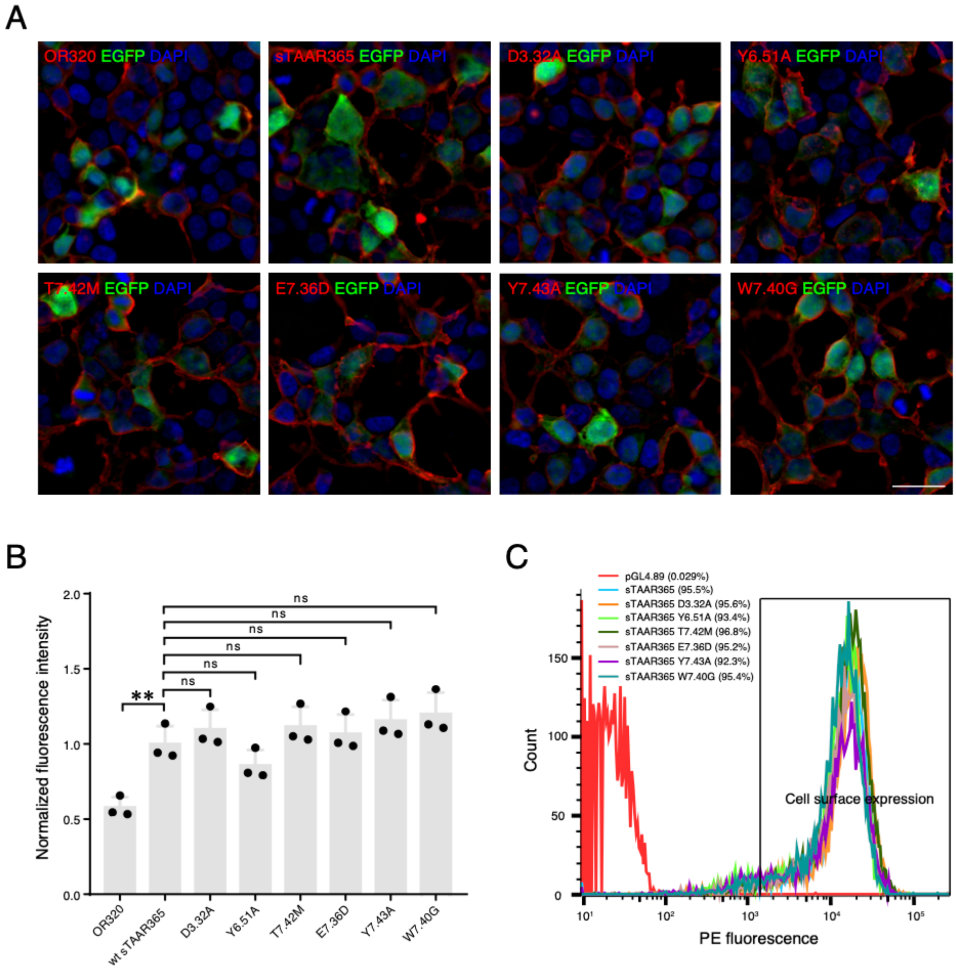

Supplementary Fig S9. sTAAR365 mutants are well-expressed on the surface of HEK293T cells with comparable expression level to wildtype sTAAR365.

(A) sTAAR365 and its mutants are well-expressed on the cytomembrane of HEK293T cells as shown by the immunostained Rho-tag antibody (red) for membrane-bound expression. HEK293T cells expressing sTAAR365, sTAA365 mutants, and OR320 were labeled with mouse monoclonal anti-Rho-tag antibody (without permeation) and incubated with red-fluorescent Alexa Fluor 594 goat anti-mouse IgG. Receptors located on the cell surface appeared as red rings around the nucleus. The nucleus was counterstained with DAPI (blue). EGFP was used as a control to evaluate transfection efficiency (green). Scale bar: 30 μm. **(B)** The mean value of red fluorescent signals of sTAAR365, mutants, and control OR320 were quantified by LAS X software (Leica). Red fluorescent intensity of sTAAR365 mutants and control OR320 was normalized to fluorescence value of wildtype sTAAR365. All mutants showed no significant difference in membrane expression level from wildtype sTAAR365 (mean ± S.D., n = 3; One-way ANOVA, $p > 0.25$). **(C)** HEK293T cells expressing sTAAR365 and sTAAR365 mutant receptors were labeled with mouse monoclonal anti-Rho-tag antibody (without permeation) and incubated with phycoerythrin-conjugated donkey anti-mouse IgG to perform flow cytometry analysis. The numbers inside the parentheses represent the percent of phycoerythrin positive cells among EGFP positive cells. D3.32A, Y6.51A, T7.42M, E7.36D,

Y7.43A, and W7.40G mutants showed comparable cell-surface expression level to wildtype sTAAR365.

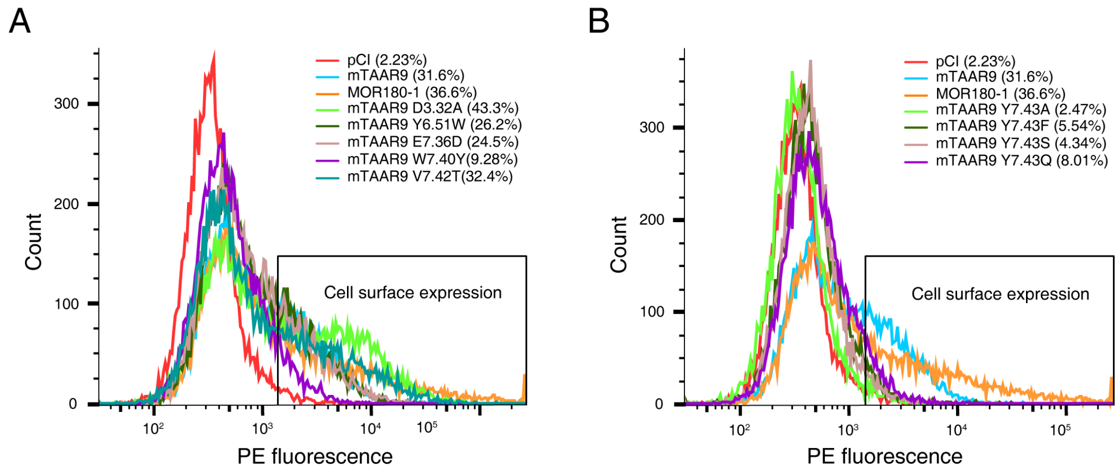

Supplementary Fig S10. Flow cytometry analysis of mTAAR9 and its mutants.

Hana3A cells expressing mTAAR9, mTAA9 mutants, and controls were labeled with mouse monoclonal anti-Rho-tag antibody (without permeation) and incubated with phycoerythrin-conjugated donkey anti-mouse IgG to perform flow cytometry analysis. The numbers inside the parentheses represent the percent of phycoerythrin positive cells among EGFP positive cells. **(A)** D3.32A, Y6.51W, E7.36D, and V7.42T mutants showed comparable cell-surface expression level to wildtype mTAAR9. W7.40Y mutant showed significant decrease in cell-surface expression level compared to wildtype mTAAR9. **(B)** Y7.43A, Y7.43F, Y7.43S, and Y7.43Q mutants showed drastic decrease in cell-surface expression level compared to the wildtype mTAAR9.
